# Immunoinformatic Prediction of HIV-1 Glycoprotein gp120 and Nef Epitopes Conjugated to HBsAg-Binding Protein (SBP) to Induce the Humoral and Cellular Immune Response

**DOI:** 10.3390/ijms26199828

**Published:** 2025-10-09

**Authors:** Arslan Habib, Xinyi Xu, Jun Xie, Naishuo Zhu

**Affiliations:** 1 Laboratory of Molecular Immunology, State Key Laboratory of Genetic Engineering, School of Life Sciences, Fudan University, Shanghai 200433, China; 20110700169@fudan.edu.cn (A.H.); 18110700002@fudan.edu.cn (X.X.); 2 Institute of Biomedical Sciences, School of Life Sciences, Fudan University, Shanghai 200438, China

**Keywords:** bioinformatics, Gp120, HIV-1, immune system, Nef

## Abstract

Acquired Immunodeficiency Syndrome (AIDS) is caused by Human Immunodeficiency Virus (HIV), and continues to be responsible for a substantial number of deaths worldwide each year. Development of a robust and efficient HIV-1 vaccine remains a critical priority. Structural analysis of viral proteins provides a foundational approach to designing peptide-based immunogenic vaccines. In the current experiment, we used computational prediction approaches alongside molecular docking and molecular dynamics (MD) simulations to identify potential epitopes within gp120 and Nef proteins. The selected co-epitopes were fused with the HBsAg-binding protein (SBP), a 344-amino acid protein previously identified in our laboratory through screening of a human liver cDNA expression library against HBsAg, to facilitate efficient delivery to and uptake by dendritic cells (DCs), thereby enhancing antigen (Ag) presentation. Flexible linkers are used to connect B cells, Helper T Lymphocytes (HTLs), and Cytotoxic T Lymphocytes (CTLs) in a sequential manner. The assembled vaccine construct comprises 757 amino acids, corresponding to a recombinant protein of 83.64 kDa molecular weight. Structural analysis through docking studies, MD simulations, and 3D structure validation revealed that the designed protein exhibits high structural stability and potential for interaction with Toll-like receptors (TLRs). These findings support the vaccine’s ability to enhance cellular and humoral feedback, including the stimulation of T and B cells and induction of antibody (Ab) production. The results underscore the promise of this in silico designed co-epitope vaccine as a viable candidate for HIV-1 prevention and suggest that such constructs may serve as effective immunogens in future HIV-1 vaccine strategies.

## 1. Introduction

Retroviruses such as HIV-1 and HIV-2 are responsible for causing AIDS in humans [1]. Despite being distinct types, they share commonalities in replication mechanisms, modes of transmission, and clinical manifestations [2]. Of the two, around 630,000 AIDS-related fatalities occurred in 2024, with an estimated 40.8 million individuals living with HIV-1 globally. This represents a minor decline in mortality as compared to 2020, although overall prevalence is still rising [3]. Notably, more than 80% of adult HIV-1 infections are acquired through mucosal exposure, with sexual contact being the predominant route [4]. As the virus continues to spread, there is an urgent need for scientific research to develop a more potent vaccine. However, the virus’s wide genetic diversity, which results in several subtypes, makes vaccine development difficult [5]. The viral Env glycoproteins gp120 and gp41 are particularly variable and act as the key part of the virus to infect host cells. These glycoproteins facilitate viral ingress by mediating the virus attachment to the host T cells [6]. Among them, gp120 is critically involved in recognizing host receptors by binding to CD4 on T cells [7]. One proposed approach to prevent infection involves neutralizing the attachment between gp120 and the CD4 receptor, thereby inhibiting viral entry into CD4^+^ cells [8]. In this context, the generation of neutralizing Abs has emerged as a promising avenue, as these Abs can target the gp120-CD4 interface and compete with CD4 for binding [9]. Experimental studies have demonstrated that such Abs can effectively neutralize gp120, reinforcing its value as a prime target for the potent and effective HIV-1 vaccine development candidates [4].

Nef is one of the first proteins translated after HIV-1 enters the host cell. It typically consists of 200 to 215 amino acids, with the 206-amino-acid variant being the most prevalent. Nef is structurally composed of a flexible C-terminal length, a central core domain, and an N-terminal membrane-anchoring region. People with Nef-deficient HIV-1 strains have reported a noticeable slowdown in the course of their illness [10]. This highlights Nef as a key pathogenic element, notable for its diverse functions and numerous cellular interaction partners [11]. Its most well-characterized functions include enhancing viral infectivity, interfering with host cell signaling and activation, and downregulating CD4, CD8, CD3, and MHC-I molecules [12]. These multifaceted actions of Nef contribute significantly to HIV-1 pathogenesis and efficient viral replication. Additionally, Nef exhibits sequence variability across different HIV-1 isolates and undergoes frequent mutations, enabling immune evasion [13]. The importance of Nef in viral replication, immune modulation, and pathogenicity emphasizes its value in vaccine development. As a key element recognized by cellular immunity, the recognition and depiction of T cell epitopes within Nef offer promising opportunities for the development of potent therapeutic and preventive approaches [14].

A distinct group of people living in a community with HIV, known as elite controllers, possess the remarkable ability to persist symptom-free and maintain elevated CD4^+^ T cell levels over an extended duration without the aid of antiretroviral therapy (ART) [15]. Studies have shown that these individuals exhibit increased populations of CTLs capable of producing interferon-gamma (IFN-γ), which promotes Th1-mediated immune feedback, suggesting its involvement in effective HIV-1 control [16]. Proliferation of CTLs has been demonstrated to significantly assist in the suppression of HIV infection [17]. Despite extensive efforts, traditional vaccine development approaches have largely failed to produce a successful HIV-1 vaccine. In contrast, a novel and promising avenue involves therapeutic immunization of people with an existing HIV-1 infection, which has shown potential in delaying or preventing progression to AIDS [18].

**Figure 1:**
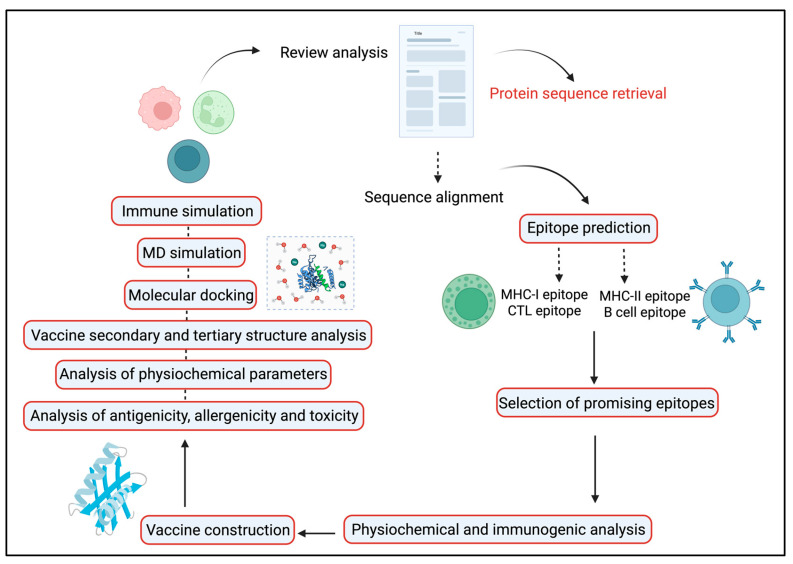
Diagrammatic illustration of the overall procedure for the final vaccine construct.

A therapeutic HIV-1 vaccine aims to trigger highly robust and comprehensive immune feedback, particularly those directed at conserved regions of the virus rather than those typically elicited during natural infection. However, the successful progress of such vaccines has been hindered by issues such as inefficient delivery systems and inadequately optimized immunogen designs [19]. An emerging and promising approach in this area involves constructing simulated multi-epitope Ags. These are engineered by selecting epitopes from common viral immunogens and incorporating a broad spectrum of protective, immunoregulatory T cell epitopes (Figure 1). The goal is to induce potent and targeted immune feedback capable of effectively controlling HIV-1 infection [20]. To subdue initial restrictions and enhance the efficacy of vaccines, researchers are turning to computational tools. In silico techniques enable the identification of potentially immunogenic peptide sequences (epitopes) from linear protein sequences. Additionally, MD simulations are used to assess the binding affinities and determine epitope MHC complexes, helping to distinguish the most promising epitope candidates [20]. Rodríguez-Fonseca and colleagues used three-dimensional (3D) models of the gp120 protein to study dendrimer-G4-PAMAM-peptide complexes. Sole peptides or the peptide-dendrimer formulated mixtures were intravenously administered to female BALB/c mice in the current experiment. Their findings demonstrated that the peptides induced immune feedback at both systemic and mucosal regions. Notably, the serum and nasal secretions resulted in elevated levels of IgG and IgA Abs after dendrimer-peptide formulated vaccine administration [21]. As the gp120 protein has a pivotal role in viral attachment to its host receptor and pathogenicity so we selected this Ag as a principal model in our HIV-1 vaccine development program. This study aimed to develop a co-epitope vaccine capable of eliciting strong T cell–mediated immunity, with emphasis on CTLs and HTLs. Using computational biology methods, we identified and validated conserved antigenic epitopes from the gp120 and Nef proteins of HIV-1, selected for their potential to activate both B- and T-cell responses. These epitopes were combined into a final vaccine construct using suitable linkers and the SBP adjuvant to enhance cellular immune activation.

## 2. Results

The primary objective of this computational-based analysis is to find the most immunogenic epitopes from the gp120 and Nef proteins to further perform in vitro and in vivo experiments. Collectively, full-length 115 HIV-1 gp120 and Nef protein full sequences were obtained. In order to produce a consensus sequence for additional study, these sequences were aligned using several sequence alignment techniques. The antigenic potential was analyzed by employing the VaxiJen v2.0 tool. Proteins’ transmembrane helices were analyzed by using the TMHMM v2.0 tool, and results showed that the number varied between 0 and 1. To assess the allergic reaction from protein sequences, we used the AllergenFP v1.0 tool, which is very commonly used in this kind of prediction. We computed various metrics such as GRAVY, theoretical pI, and half-life scores in order to evaluate the vaccine construct’s antigenicity as well as other physicochemical characteristics [22].

The protein reaches its zero-charge point when the pH equals the theoretical pI value. The protein structure demonstrates elevated aliphatic indices because of its high score of aliphatic amino acid residues in the side chains. The GRAVY value demonstrates whether the protein is hydrophobic or hydrophilic. Proteins with negative GRAVY scores dissolve well in water, and proteins with positive scores exhibit insolubility [23]. Finally, epitope predictors were applied to the consensus sequence, leading to the recognition of several peptides. Table 1 mentions all the computational tools used in this study.

### 2.1. Linear and Conformational B Cell Epitope Assessment

**Figure 2:**
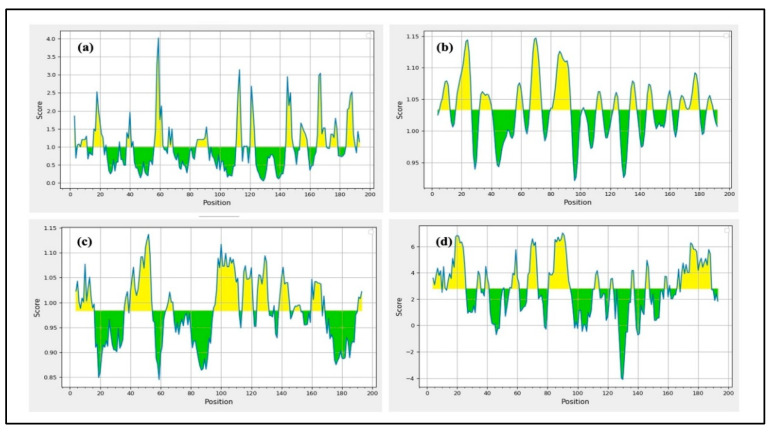
B cell epitopes were selected by several IEDB epitope servers. Yellow highlights peptides that show favorable interactions. (**a**) The Emini server was used to predict the surface proximity of B cell epitopes. (**b**) The Karplus & Schulz method was used to assess the flexibility of a few chosen epitopes. (**c**) The Kolaskar & Tongaonkar approach was used to assess the final epitopes antigenic potential. (**d**) The Parker approach was used to analyze the hydrophilicity.

**Figure 3:**
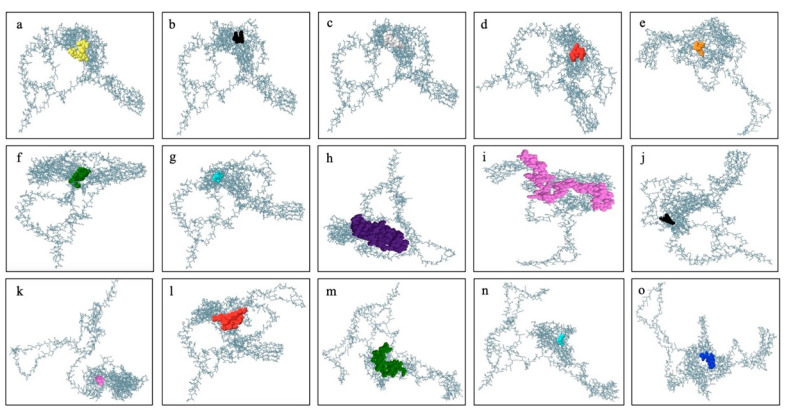
The final vaccine design shows the conformational or discontinuous epitopes. Predicted conformational B-cell epitopes on the vaccine construct. Epitope prediction was performed using the ElliPro tool. (**a**–**o**) present in the highest antigenic polyprotein of gp120 and Nef in 3D format. The proportions of the polyprotein are mentioned by sky blue sticks, whereas the epitopes are shown on the surface using different colors.

Initially, IEDB and BepiPred 2.0 were used to perform the epitope predictions, and BepiPred predictions were verified using the iBCE-EL program [24]. The iBCE-EL-identified epitopes that the other three servers failed to detect were eliminated. B cell peptides are placed in solvent-favored areas of the Ag to promote contact with B cells and guarantee their effectiveness. Predicting the protein sequence’s surface accessibility was therefore essential. The HIV-1 gp120 and Nef protein sequences’ surface accessibility were analyzed by the Emini server (Figure 2), and non-surface-exposed epitopes were eliminated. From the chosen proteins, 16 putative linear B cell peptides were found (Table 2). The Karplus & Schulz, Kolaskar & Tongaonkar, and Parker techniques were used to estimate the discovered epitopes flexibility, antigenicity, and hydrophilicity, respectively (Figure 2). The variation in protein structure and folding processes leads to the development of distinct conformational B cell epitopes. The ElliPro program processed the modified 3D structures to identify the conformational B cell epitopes. The investigation exposed 16 potent conformational B cell epitopes comprising 757 residues, which achieved scores between 0.525 and 0.786. Figure 3 shows a 3D representation of the conformational epitopes in the final vaccine design.

### 2.2. CTL Epitope Prediction Selection

Through their interactions with MHC-I molecules, which bind to particular CTL epitopes, CTLs significant role in the detection of infected cells. The IEDB prediction technique was assessed to anticipate MHC-I-binding epitopes in the gp120 and Nef proteins. Based on the significant immunogenicity and antigenicity of the chosen proteins, 66 identified 8- to 12-mer CTL epitopes were found (Table 3 and Table 4). Furthermore, these epitopes to bind mouse MHC-I alleles were assessed. The epitope candidates underwent additional evaluation based on immunogenicity, worldwide population coverage across various locations, and mean MHC-I processing values. Following that, the epitopes with the significant values were analyzed for possible allergenicity, toxicity, and chemotoxicity as well as for conservancy across different HIV-1 clades. Additionally, as the IEDB tool anticipated, MHC-I epitopes were selected due to their increased binding affinity. The final selection procedure did not include any overlapping MHC-I epitopes.

### 2.3. CTL Immunogenicity Assessment

The European Molecular Biology Open Software Suite (EMBOSS 6.4.0) and the IEDB server were used to forecast the immunogenicity scores for the chosen MHC-I epitopes as well as the manipulation of T cell epitopes. According to the IEDB immunogenicity tool, the epitopes with significant immunogenicity values revealed by the analysis were selected. To determine which epitopes were most likely to induce a robust immune response, the prediction algorithm considered both the immunogenicity and antigenicity ratings.

### 2.4. HTL Epitope Selection

By connecting with particular HTL epitopes on MHC-II molecules, HTLs are able to identify infections and assist the activity of other immune cells. The peptides were transmitted to the IEDB website in order to evaluate their binding to MHC-II alleles in the gp120 and Nef Ags. Out of the chosen proteins, this analysis found 40 putative HTL epitopes (Table 5 and Table 6). The predicted epitopes vary in size from 14 to 16 amino acids and show high binding affinities for a significant number of HLA-II alleles. The predicted population coverage of these HTL epitope candidates in different parts of the world was then used to filter them. Properties including allergenicity, toxicity, chemotoxicity, their potential to induce cytokine induction, and their conservation across several HIV-1 clades were further evaluated for the top-scoring epitopes. Overlapping MHC-II epitopes were handled as separate entities. All MHC-II epitopes were assessed for their potential to promote IFN-γ secretion in order to finalize the screening process.

### 2.5. Most Effective Epitope Selection

To construct the vaccine, both B and T cell epitopes were evaluated based on their physicochemical properties. HTLs contribute to the activation of B cells, macrophages, and CTLs, among other immune cells, whereas CTLs are in charge of identifying Ags [25]. B cells can develop into plasma cells, which provoke the humoral immune response by developing Abs. Nevertheless, the humoral immune feedback is less efficient than the cell-mediated immune response and deteriorates in the course of time [26]. By producing antiviral cytokines and particularly recognizing and eliminating infected cells, the cell-mediated immune response, on the other hand, offers more robust and long-lasting protection. Nef and gp120 proteins served as models for epitope discovery in this investigation. Numerous epitopes were found through the analysis, but the most potent T cell epitopes were selected for subsequent study. For further screening, B cell epitopes longer than 7 amino acids were also given priority. After evaluating several criteria, such as high antigenicity, non-allergenic and non-toxic characteristics, strain-to-strain conservation, and little resemblance to the human proteome, the B and T cell epitopes were selected. The epitopes that satisfied these requirements were included in the production of the final vaccine construct. Only HTL epitopes with the capacity to induce cytokine production were included in the vaccine development process after their potential was assessed.

### 2.6. Proteasomal Cleavage/TAP Transport

Proteasomal cleavage and TAP transport play crucial roles in the Ag presentation process, so it was imperative to identify the gp120 and Nef Ags using the NetCTL1.2 servers [27]. Table 3 and Table 4 display the TAP transport and proteasomal cleavage scores for every CTL epitope. The CTL epitopes with the highest immunogenic scores were chosen for the final vaccine design.

### 2.7. Epitopes Inducing IFN-γ

IFN-γ is essential for antiviral defense because it activates NK cells and macrophages, boosting both the innate and adaptive immune system. Additionally, IFN-γ enhances MHC molecule activation in response to Ags. HTL epitopes were ultimately chosen based on their capacity to stimulate the generation of IFN-γ and their potential for binding MHC-II, which is imperative for HTL activation. During the screening phase, a number of epitopes were eliminated because they were unable to cause the secretion of IFN-γ [28]. The 40 HTL epitopes that were favorably predicted for both immunogenicity and IFN-γ induction are included in Table 5 and Table 6. Similarly, epitopes that did not occur in this group but were anticipated to have negative IFN-γ release were also eliminated.

### 2.8. Population Coverage Prediction

**Figure 4:**
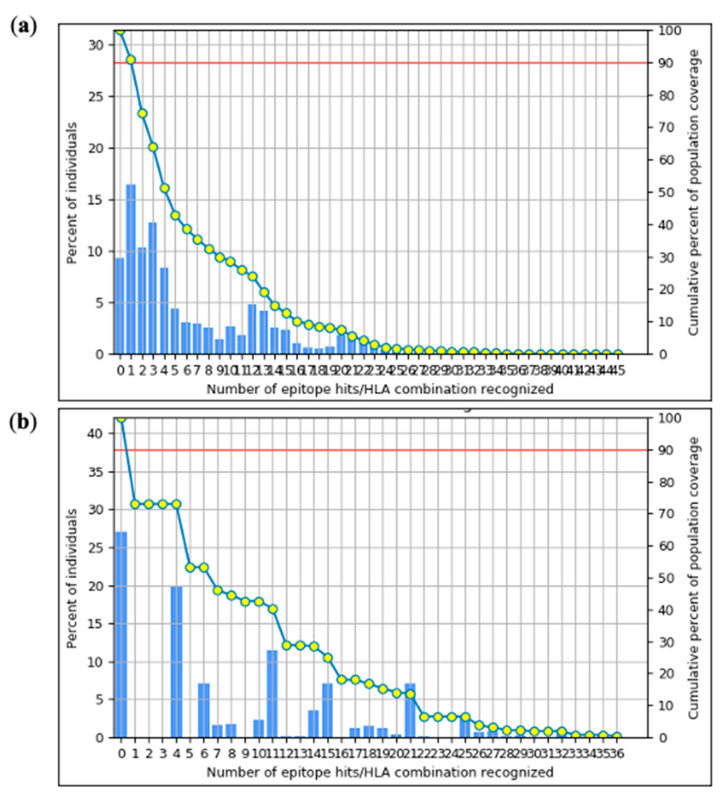
Both global and average percentages were taken into account when calculating the population coverage (%) associated with the HLA binding alleles of the chosen epitopes. (**a**) The chosen epitopes for MHC-I restricted alleles have, therefore, a population coverage of 90.76% worldwide. (**b**) On the other hand, the chosen epitopes for MHC-II restricted alleles account for a population coverage of 73.35% worldwide.

The global coverage of the chosen epitopes within the organized sequences was analyzed using the IEDB Population Coverage application. According to the analysis findings, the worldwide population was covered by about 90.76% of the epitopes connected to MHC-I alleles and 73.35% by those linked to MHC-II alleles. MHC-I/II epitopes of the gp120 and Nef Ags had the maximum population coverage as illustrated in Figure 4. These two classes, I and II representations, collectively provide a predicted population coverage of 96.23%, notable for its global potential. According to this population coverage analysis, the target MHC alleles are present in a significant fraction of the world population [29]. As a result, we anticipate that the produced vaccine will effectively tackle the virus on a global scale.

### 2.9. Antigenicity, Allergenicity, and Solubility Analysis

B cell, CTL, and HTL epitopes’ antigenicity determinations included the use of Vaxijen 2.0 and AntigenPro servers. Epitopes considered to have high antigenic potential were chosen for the final vaccine construct. Only those epitopes were considered for the vaccine that were non-allergenic; the servers used for the allergenicity prediction were AllergenFP 1.0 and AllerTOP 2.0. Solubility of each epitope was predicted based on two servers, SolPro and Protein-sol. The final design of epitopes was performed guided by solubility criteria so that the most favorable solubility profile could be chosen. Table 2, Table 3, Table 4, Table 5 and Table 6 display the immunogenic parameter data for each epitope.

### 2.10. Toxicity and Physicochemical Characteristics Analysis

Determining how the vaccine will interact with the body physiology requires assessing its lack of toxicity and comprehending its physicochemical characteristics. The ToxinPred server was used to forecast toxicity. Numerous physicochemical characteristics, including hydropathicity, charge, half-life, instability index, pI, and molecular weight, were also predicted using the ExPASy ProtParam Tool. Overlapping peptides from B cell, CTL, and HTL epitopes, each having a maximum length of 20 amino acids, were submitted to ToxinPred. After a series of evaluation tests, non-toxic peptides were identified and incorporated into the final vaccine design. The entire vaccine sequence, including the adjuvants, was screened to check for the existence of any other toxic peptides. The rest of the subunits and the HisTag were further analyzed using ToxinPred SVM prediction mode to confirm their nontoxic status. The hydropathicity of each epitope was also predicted, and only those with negative values, suggesting that the epitopes are hydrophilic and easily interact with water molecules, were selected. Table 2, Table 3, Table 4, Table 5 and Table 6 display the findings for each epitope’s physicochemical characteristics and toxicity.

### 2.11. MHC Restriction of Epitopes and Cluster Evaluation

**Figure 5:**
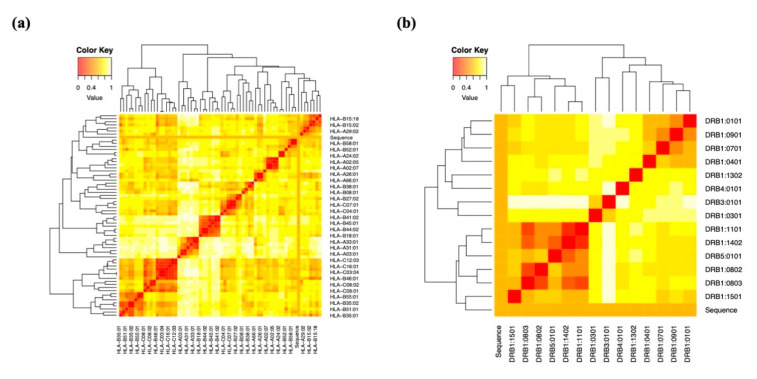

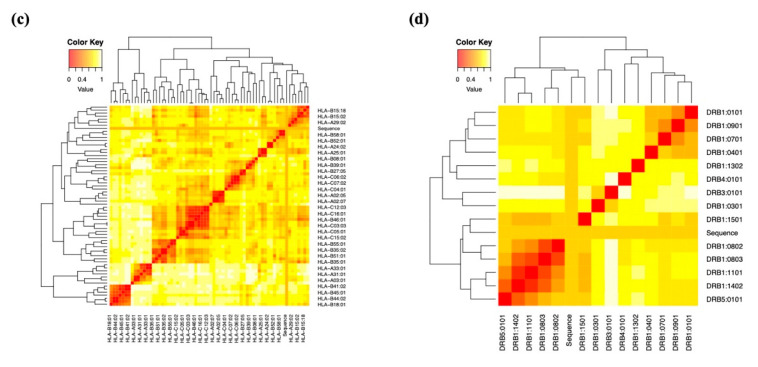
Heat map representation in cluster analysis of HLA alleles for both MHC molecules. (**a**,**b**) Cluster analysis of the MHC-I and MHC-II molecules was carried out using the most promising gp120 epitopes used in the final vaccine construct. (**c**,**d**) Cluster analysis of the MHC-I and MHC-II molecules was carried out using the most promising Nef epitopes used in the final vaccine formulation.

Following the physicochemical investigation, MHC cluster analysis was used to confirm that the chosen epitopes were MHC-restricted. Heat maps for MHC classes I and II were created for gp120 using the results of the evaluation of the interacting alleles (Figure 5a,b). Heat maps for Nef were also generated by using the same method (Figure 5c,d). The epitopes were grouped based on how well they correlated with HLA; weak connections were shown in yellow, and strong interactions in red.

### 2.12. Modeling of Co-Epitope Subunit Vaccine

**Figure 6:**
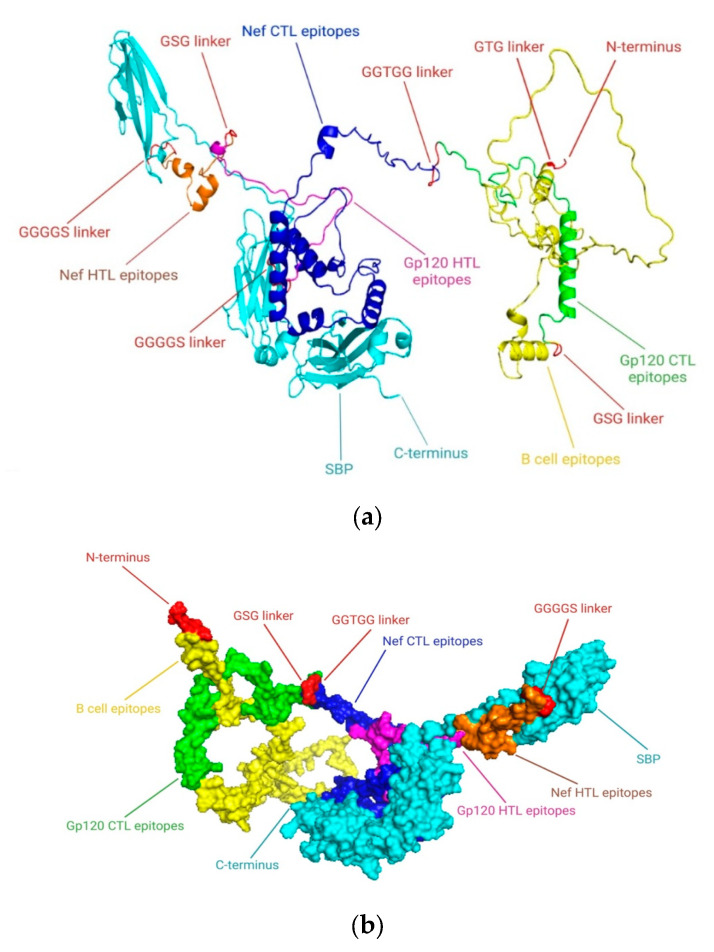
The final vaccine construct’s immunodominant B cell, CTL, and HTL epitope sequences, locations, and representations are displayed along with the 3D site and positions of the immunodominant gp120 and Nef epitopes predicted by RoseTTAFold. (**a**) A cartoon form represents the immunodominant epitope locations and sequences. (**b**) PyMOL 2.3.4 is used to represent the immunodominant epitopes in a 3D format. The positions of the immunodominant epitopes on the closed geometry of the 3D final vaccine construct are displayed in the surface representation.

**Figure 7:**
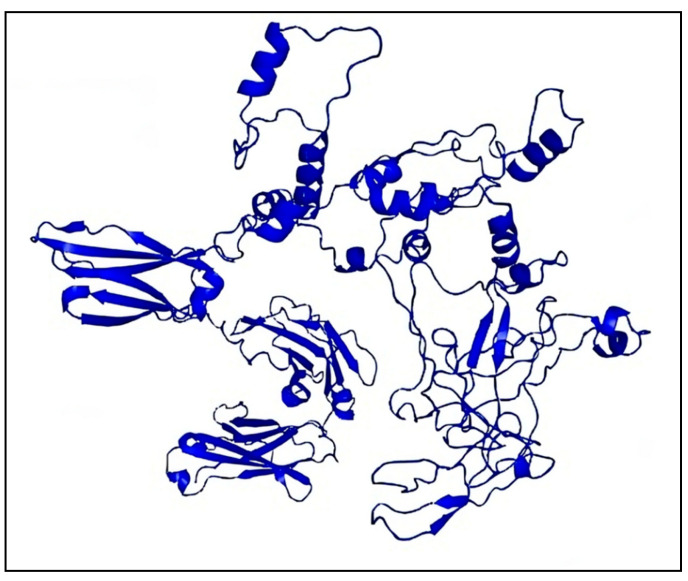
SBP 3D model designed using RoseTTAFold software.

According to “Selecting the Most Promising Epitopes”, epitopes that were found to have high antigenicity, no toxicity or allergens, conservation across specific strains of gp120 and Nef, the capacity to induce cytokine production, especially for HTL epitopes, and low resemblance to the human proteome were used for final vaccine development. These epitopes were thought to be the best fit for the vaccine’s formulation. To efficiently stimulate the innate immune system and elicit an immunological response. In order to construct a vaccine based on epitopes, the viral proteins Nef and gp120 were examined in this study. Despite the fact that antimicrobial peptides (AMPs) are known to influence both innate and adaptive immunity, we particularly focused on viral protein epitopes in this study. Based on various immunoinformatic analyses, a total of 8 B cell, 4 HTL, and 10 CTL epitopes were chosen for the co-epitope vaccine. Figure 6 displays the final immunodominant epitopes in a 3D model. The final epitopes were linked with particular linkers: the B cell epitopes were linked with the GTG linker, and the B cell and CTL epitopes were linked with the GSG and GGTGG linkers. The HTL and CTL epitopes were joined by a GGGGS linker, while the C-terminal of the SBP was joined by a GGGGS linker. A vaccine design of 757 amino acid residues was produced after the appropriate combination and randomization. Figure 7 displays the 3D structure of the SBP.

### 2.13. Antigenicity, Allergenicity, and Physicochemical Parameters of Vaccine Formulation

Computational analyses predicted that the HIV-1 gp120-Nef vaccine construct possesses high antigenicity and is non-allergenic based on antigenicity and allergenicity prediction tools, indicating its capacity to provoke a robust immune response without producing unfavorable allergy reactions. The suitability of the vaccine for development was next confirmed by an evaluation of its physicochemical characteristics. The formed gp120-Nef construct had the chemical formula C_3701_H_5680_N_1044_O_1124_S_27_ and a molecular weight of 83.64 kDa. The vaccine stability at an adult’s normal body temperature was shown by the aliphatic index (AI), a thermostability metric, which was 59.52. The vaccine’s alkaline nature was revealed by its theoretical pH of 8.87. The vaccine proteins appear to be hydrophilic based on the negative GRAVY score of −0.646. ProtParam estimated a half-life of more than 10 h in *E. coli* culture and 30 h in human reticulocytes, suggesting stability across expression systems. Furthermore, SOLpro analysis predicted that the gp120-Nef vaccine would likely be highly soluble upon expression in *E. coli*, supporting its potential suitability for large-scale production and purification. Since insoluble recombinant proteins may cause improper folding or form insoluble inclusions, rendering them nonfunctional, solubility is a crucial consideration in post-production processing. Furthermore, during vaccine purification, excellent solubility makes purifying easier [30]. All parameters were considered; these outcomes signify that the suggested vaccine formulation may be a useful construct for HIV-1 prevention. Table 7 provides a summary of the formulated vaccine design’s physicochemical characteristics.

### 2.14. Assessment of the Secondary Structure of the Vaccine Construct

**Figure 8:**
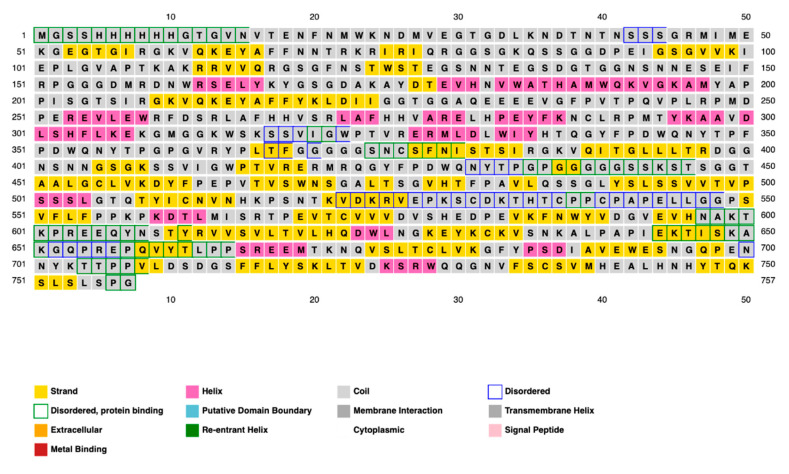
The final vaccine construct’s secondary structure features are represented graphically. The residues of the coil are gray, those of the alpha helix are pink, and those of the beta strand are yellow. The final vaccine construct has 59.84% random coils, 29.33% β-strand, and 10.83% α-helix according to an exploration of the projected secondary structure.

**Figure 9:**
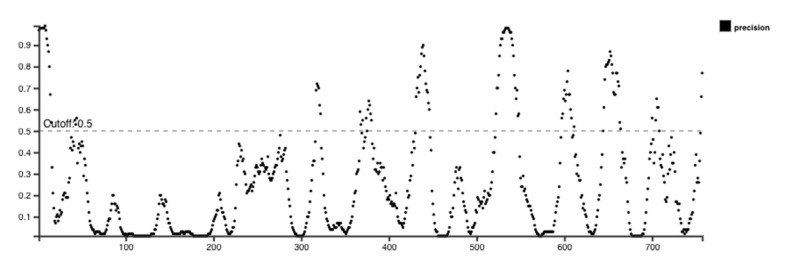
The PSIPRED tool was used to forecast the disorder regions in the secondary structure design. It has specifically located disordered areas in the protein sequence. The analysis indicates that when the dotted line surpasses the cutoff value of 0.5, which is the confidence level, amino acids in the input sequence are deemed disordered.

The distribution of amino acid structured elements, such as α-helix, β-strand, and coil in the vaccine construct was studied by three different prediction methods, such as the PSI-blast secondary structure prediction server, PSIPRED, SOPMA, the Self-Optimized Prediction Method With Alignment, and Phyre2 servers. The results showed that the distributions of amino acids between the structures most and least frequently found varied significantly, while the α-helix, β-strand, and coil structures showed consistent results on all three servers used. The final assessment was conducted mostly using the PSIPRED software (Version 4.0). The features of the secondary structure are illustrated graphically in Figure 8. The final vaccine construct has 59.84% random coils, 29.33% β-strand, and 10.83% α-helix, according to the SOPMA server’s analysis. Additionally, 16% of the regions were found to be disordered by the secondary structure analysis (Figure 9).

### 2.15. Modeling of 3D Vaccine Construct Improvement and Confirmation

**Figure 10:**
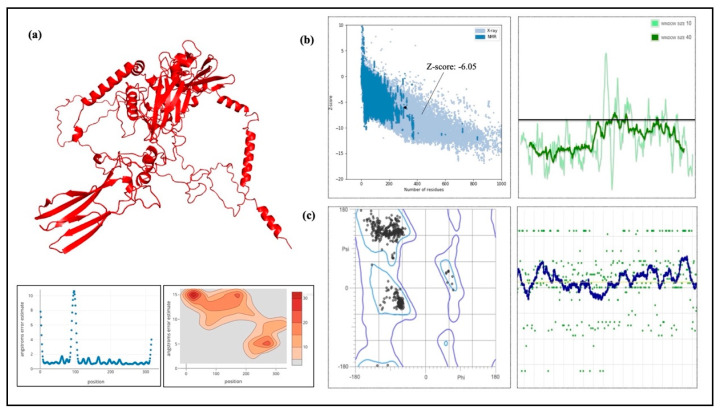
HIV-1 final vaccine representation and validation with in silico technologies. (**a**) Galaxy Refine was used to model the final vaccine construct in 3D using fine-tuned variables. (**b**) Prosa-web validated our 3D vaccine construct by producing the Z-score and local model quality energy value. (**c**) The accuracy of our vaccine design was further confirmed by the Ramachandran plot, which is connected to the vaccine 3D structure after refinement. Furthermore, Verify 3D was used to confirm the 3D prediction, showing that the vaccine structure obtained an impressive residue score of 91.60%.

RoseTTAFold was used to simulate the final vaccine 3D structure, which was then improved for structural stability and stereochemical quality. Figure 10 displays the vaccine design’s anticipated 3D structure. The GalaxyRefine tool was used to refine the structure, and among the five refined models generated, Model 1 showed the highest quality and was selected as the final vaccine model. The refined structure achieved a Global Distance Test-High Accuracy (GDT-HA) score of 0.9595 and a Root Mean Square Deviation (RMSD) of 0.403, indicating structural stability (values < 1.2 are generally considered favorable). The MolProbity score of 2.108 further confirmed the improved stereochemical quality of the refined model, consistent with high-quality structural models. Additionally, the refinement clashscore was reduced to 14.1, reflecting a decrease in unfavorable steric overlaps and improved overall model stability. Furthermore, after refining the Ramachandran plot, which evaluates the energetically favored regions improved from 93.5% to 96.9%. In general, a Ramachandran plot value of more than 85% is regarded as satisfactory. ProSA-web was assessed to confirm the final model, and the Z-score that was obtained was −6.05, which is within the optimum range for native proteins of corresponding size (Figure 10b). Proteins that have been modeled using X-ray or NMR methods are frequently validated using the Z-score. X-ray techniques are used to study proteins with more than 200 amino acids, while NMR is usually used to analyze proteins with fewer than 200 amino acids. Additionally, ProSA assesses the quality of the local model by displaying residue values; negative values signify that the structure is error-free. The trustworthiness of the model is further supported by the finding that 96.9% (594/757) of the residues were in favored regions as shown by a Ramachandran plot study (Figure 10c).

### 2.16. Vaccine Disulfide Bond Engineering Design

**Figure 11:**
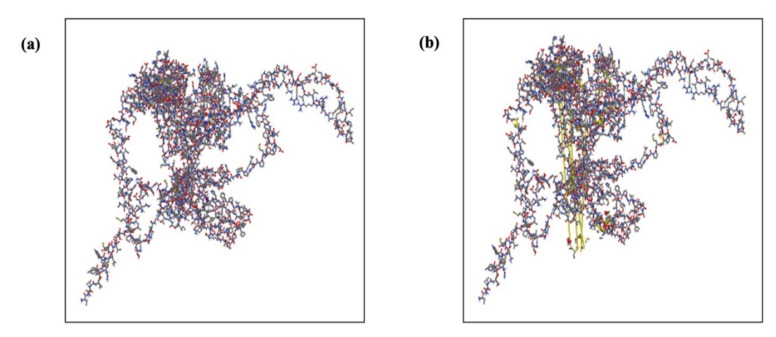
Disulfide bond engineering ensures the vaccine stability. (**a**) There are no mutations in the vaccine construct initial form. (**b**) Four pairs of amino acids, including Phe54-Gly125, Cys276-Pro310, Leu381-Ser596, and Leu607-Asp610, are shown as yellow sticks in the mutant shape. These particular pairs of amino acids have been altered to include disulfide bonds.

Overall, 36 possible pairs of amino acid residues were found to be disulfide engineering candidates using the Disulfide by Design v2.12 web server. The χ3 angle and energy score of these couples were used to further evaluate them. Following a comprehensive analysis, only four couples met the requirements: the energy score had to be less than 2.2 kcal/mol, and the χ3 angle had to be between −87° and +97°. Consequently, 4 residue pairs Phe54-Gly125, Cys276-Pro310, Leu381-Ser596, and Leu607-Asp610 were mutated. These mutations had energy values of 3.91, 5.52, 4.92, and 5.24 kcal/mol, respectively, and corresponding χ3 angles of −63.56°, +103.17°, +89.94°, and +105.83° (Figure 11).

### 2.17. Vaccine Construct’s Molecular Docking with TLRs

**Figure 12:**
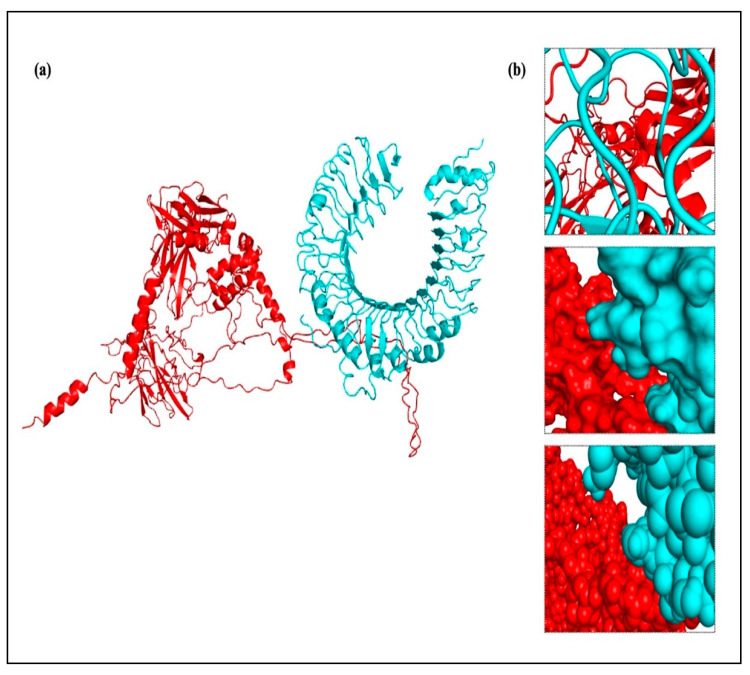
Molecular docking between the formulated vaccine and the TLR-2 receptor. (**a**) The cartoon of the vaccine TLR-complex is rendered with the red color symbolizing the vaccine complex and the cyan the TLR unit. (**b**) PyMOL presents various views of the interaction of TLR-2 and the vaccine complex. To highlight the distinction between their functionalities at the binding interface, the interaction residues of TLR-2 and the vaccine complex are proposed in different representations.

**Figure 13:**
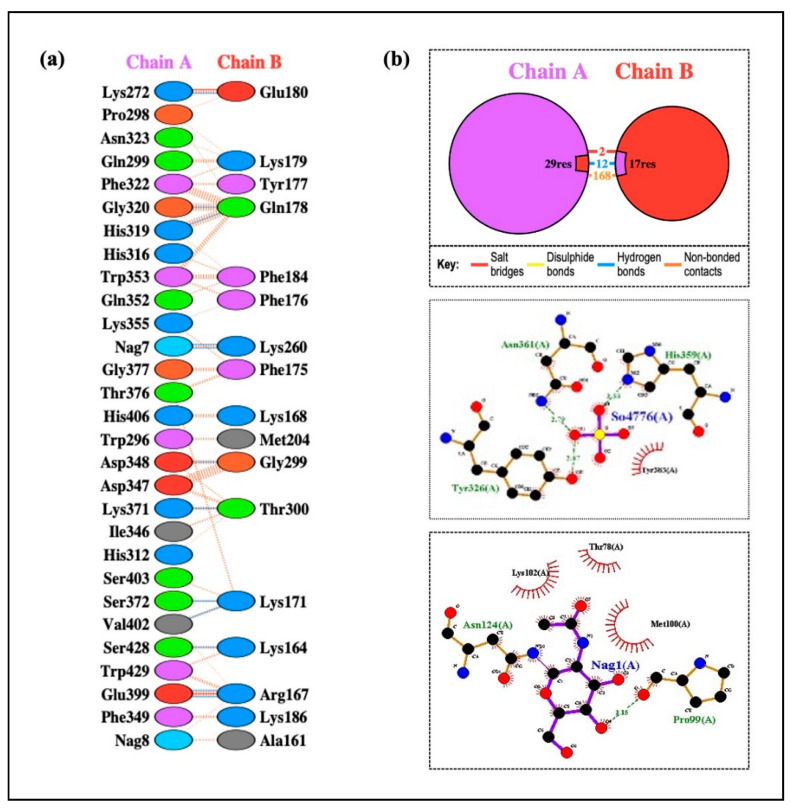
This figure depicts the binding interface in vaccine design with the active residues of the docked complex involving human TLR-2. (**a**) The active residues of TLR-2 are termed chain A for this molecule, and active residues of the vaccine construct are termed chain B. (**b**) Ligplot analysis depicts the docked conformation and interaction of TLR-2 and vaccine construct, conferring the presence of hydrophobic and hydrogen bond contacts between the constructs.

To produce a dependable and efficient immune response, it is crucial to comprehend the connection between immune cells and vaccine design. A strong technique for determining the stability and binding potential of a ligand and its receptor molecule is molecular docking. TLRs and vaccines work together to trigger a robust immune response, and TLR binding is a critical sign of successful infection prevention. The SBP sequence was conjugated to the vaccine C-terminal after the final vaccine construct was docked with TLR-2, TLR-3, TLR-4, TLR-5, and TLR-8. Molecular docking was performed using two different servers to cross-validate the predicted interactions, and the ClusPro results were selected for detailed analysis and presentation. In every docking trial, the docking results showed robust and advantageous interactions between the vaccine construct and the TLRs. The refined 3D structure of the vaccine was molecularly docked with TLRs using the HADDOCK and ClusPro 2.0 servers. Using the HADDOCK server, the interactions between TLRs and the vaccine construct were specifically examined. Based on the HIV-1 gp120-Nef protein complexes from HADDOCK, complexes with the highest ranks were chosen, taking into account the lowest mean RMSD values. Electrostatic bonding is crucial for the HIV-1 gp120-Nef-TLR-2 complex according to an examination of the HADDOCK output (Figure 12). Using the Pdbsum and LigPlot^+^ tools, the HADDOCK results were further verified, confirming that the best docking outcomes were chosen (Figure 13).

**Figure 14:**
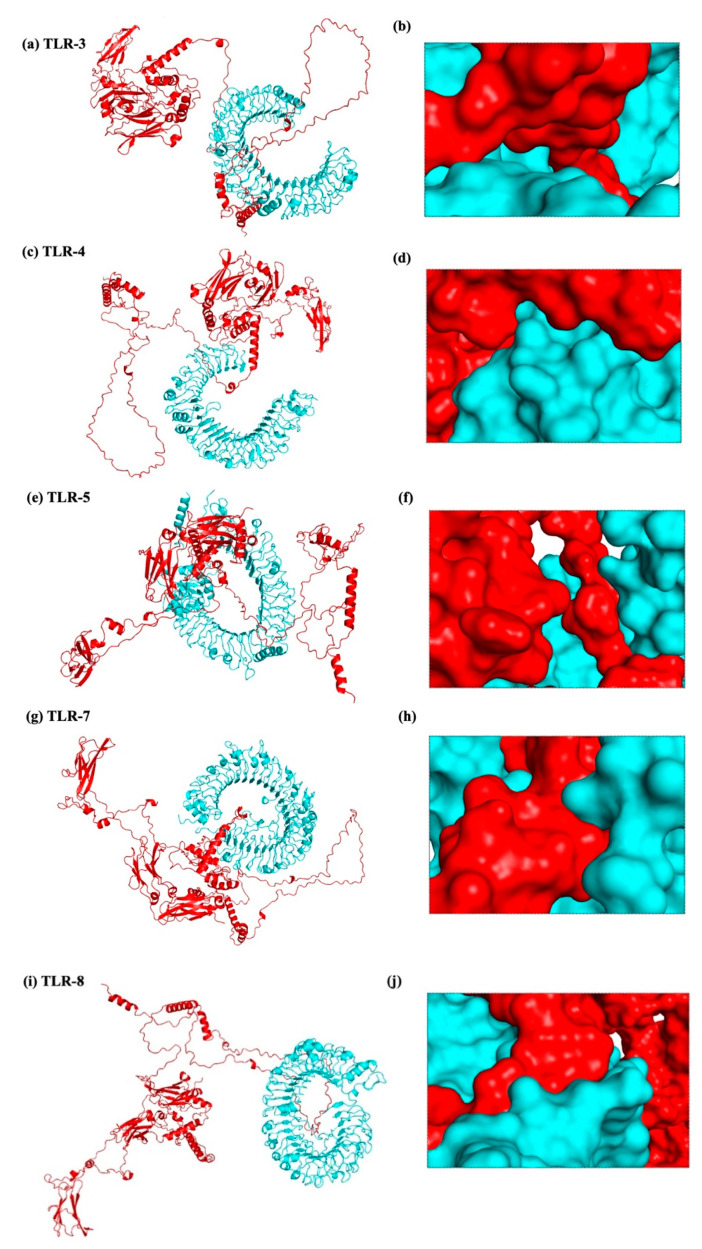
The association between the formulated vaccine construct and human TLRs was examined by molecular docking analysis. The docking areas between the vaccine construct and the TLRs were found using ClusPro. A deeper look at the docking interaction between the formulated vaccine and TLRs is shown in the figure. (**a**) represents the TLR-3 interplay with the vaccine construction. (**b**) is a closer interaction at TLR-3. (**c**) represents the TLR-4 interplay with the final vaccine construct, while (**d**) is a closer interaction at TLR-4. Similarly, (**e**) TLR-5 interplay with the vaccine construct, while (**f**) is a closer interaction at TLR-5. Further, (**g**) TLR-7 interplay with the vaccine construction, while (**h**) is a closer view of TLR-7 and (**i**) represents the TLR-8 interplay with the final vaccine construct, (**j**) shows a deep interaction of TLR-9.

**Figure 15:**
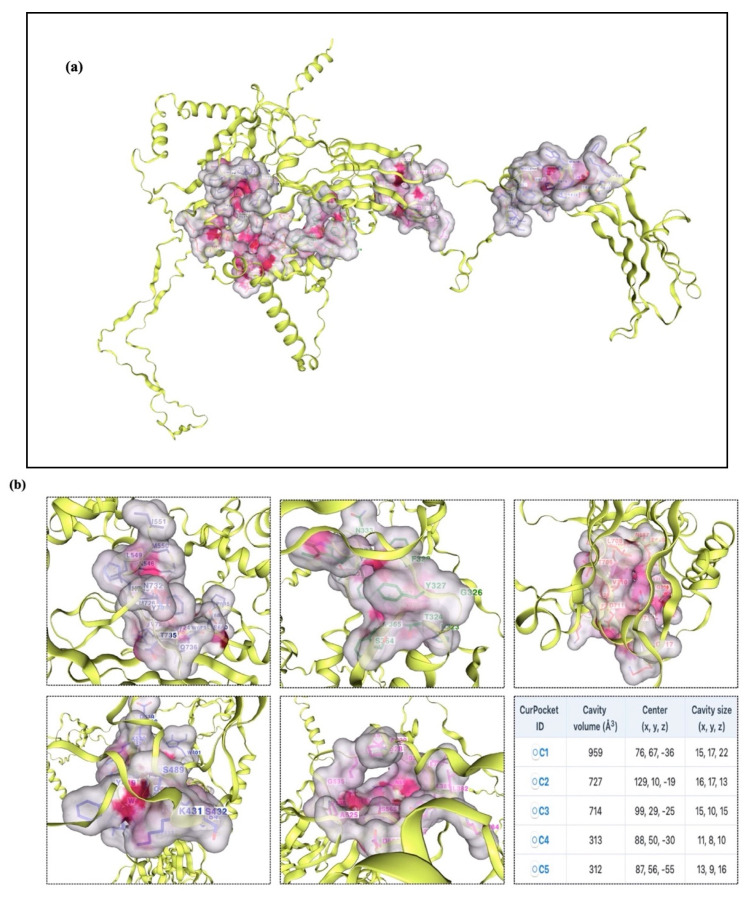
(**a**). Target protein predicted binding cavities found with CB-Dock2. The five top-ranked binding cavities (C1–C5) as predicted by CB-Dock2 are highlighted in the picture, which depicts the target protein’s 3D structure. According to its rank based on cavity volume, each cavity is labeled and color-coded. (**b**). CB-Dock2 is employed for analysis of binding cavities. Using CB-Dock2, five possible binding pockets (C1–C5) were found on the surface of the target protein. According to their volumes, the cavities are sorted with cavity C1 having the largest volume (959 Å^3^) and cavity C5 having the lowest (312 Å^3^). A list of the cavity centers’ spatial coordinates and corresponding dimensions (x, y, and z) is provided. The pockets C1, C2, and C3 exhibit the largest cavity volumes and diameters, indicating a higher possibility for ligand accommodation. These pockets are possible locations for molecular docking.

The results were further confirmed by the ClusPro docking analysis, which determined that the complex with the lowest energy was the most advantageous. TLR-2, TLR-3, TLR-4, TLR-5, TLR-7, and TLR-8 had docking energies of −1105.6, −1411.4, −1160.3, −1641.9, −1238.1, and −1317.2, respectively, with the final vaccine construct (Figure 14). Strong binding affinities in the docked complexes suggest that the vaccine efficiently interacts with TLRs. Additionally, the proposed protein was examined for possible binding cavities appropriate for docking studies using the CB-Dock2 program. Five different binding cavities with different volumes, locations, and spatial dimensions were found. These findings offer important information for choosing the best cavities for next docking research and structure-based medication design initiatives (Figure 15).

### 2.18. MD Simulation

**Figure 16:**
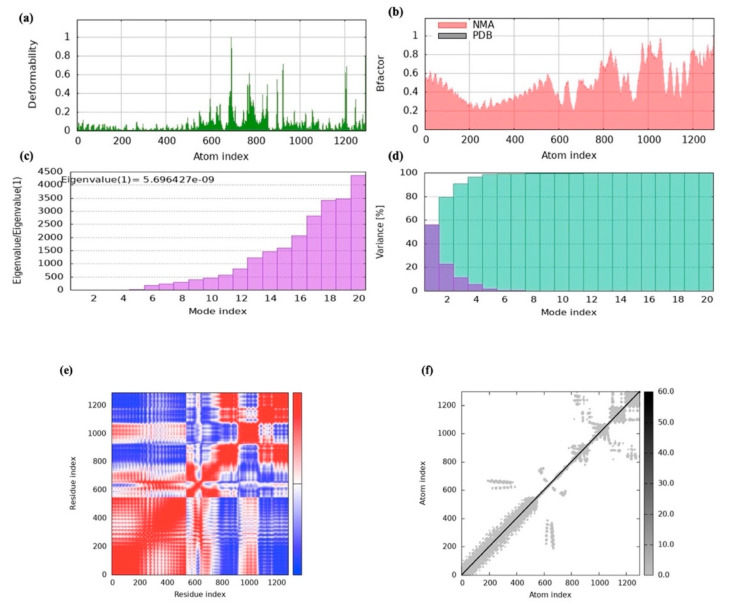
The docked complex between the vaccine and TLR-2 was simulated using MD. (**a**) To identify hinge regions with high deformability, a main-chain deformability simulation was run. (**b**) B-factor values, which provide an uncertainty metric for each atom, were computed using normal mode analysis. (**c**) The energy needed to distort the structure was stipulated by the docked complex’s eigenvalue. (**d**) The covariance matrix between residue pairs was examined and expressed by different colors. (**e**,**f**) To evoke the interactions between atoms and springs, an elastic network model was developed. Darker tones of gray indicate more rigid springs.

iMOD online tool was used to assess the dynamics and stability of the docked complex between the vaccine construct and TLR-2. Significantly flexible areas were identified by the main-chain deformability study, emphasizing the structure’s hinges (Figure 16a). The degree of atomic mobility and the uncertainty surrounding each atom are shown by the β-factor scores, which are obtained from NMA (Figure 16b). An eigenvalue of 5.696427 × 10^−9^ was found for the complex, which is linked to the energy needed for structural contortion (Figure 16c). The correlations between residue pairs are depicted in the covariance matrix, where correlated residues are shown in red, uncorrelated residues are shown in white, and anti-correlated residues are shown in blue (Figure 16d). The relationships between atoms and the forces acting on them were also visualized using the elastic network model (Figure 16e,f). The stability of our vaccine model was validated based on the outcomes of the MD simulation, demonstrating its resilience and preparedness for future advancement.

### 2.19. Codon Optimization and In Silico Cloning

**Figure 17:**
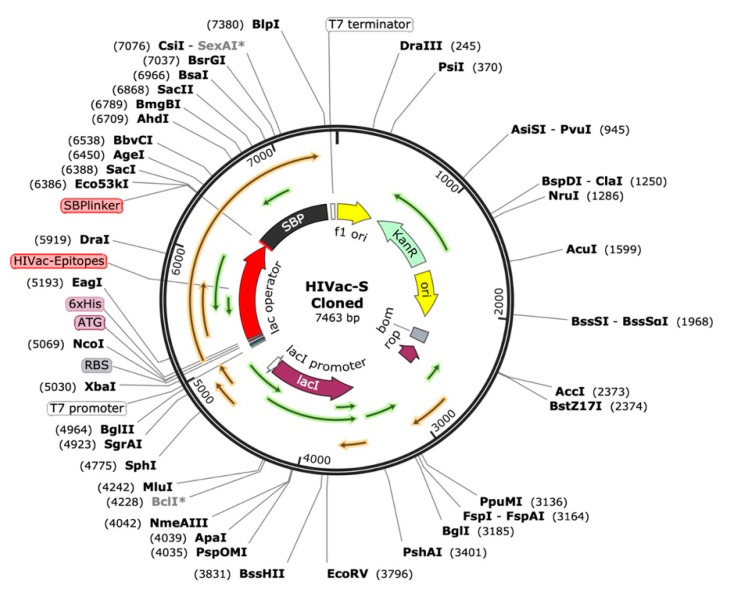
The HIV-1 vaccine construct nucleotide sequence was inserted into the pET28a vector using SnapGene. The vaccine nucleotide sequence is depicted as a red line and the SBP sequence as a black line. The *Nco*I and *Xho*I restriction enzymes are mentioned at the N- and C-terminal ends of the vaccine design, respectively. Moreover, a HisTag was included in the N-terminal site of pET28a and is illustrated as a purple box.

**Figure 18:**
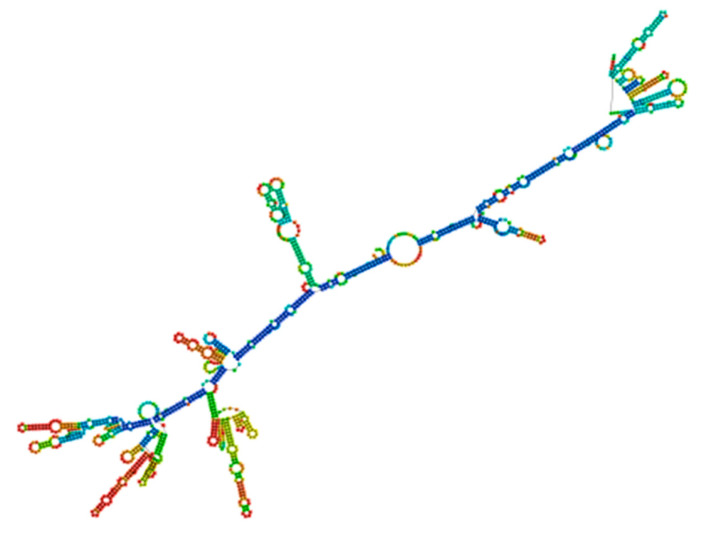
The mRNA structure of the vaccine design is depicted in this figure. The RNAfold server was used to determine the mRNA structure of the HIV-1 gp120-Nef vaccine construct. With a low free energy value of −806.89 kcal/mol, the prediction demonstrated the equilibrium of the mRNA structure of the vaccine design.

JCAT was used to optimize the nucleotide sequences for the final vaccine design. The JCAT parameters were changed to steer clear of restriction enzyme recognition regions, rho-independent transcription terminators, and bacterial ribosome binding sites. The reverse transcription-derived cDNA sequence was used for in silico cloning. The *E. coli* K12 expression system was selected. The HIV-1 gp120-Nef construct’s codon optimization study showed 54% GC content, and the gene’s strong expression potential in *E. coli* cells was suggested by the computed CAI of 0.95. A stop codon was positioned following the SBP sequence, and restriction regions for *Nco*I and *Xho*I were inserted at the N- and C-terminal of the sequence, respectively (Figure 17). RNAfold was used to analyze the vaccine’s mRNA structure, and the minimal free energy (MFE) score was −806.89 kcal/mol (Figure 18). A lower MFE score suggests enhanced mRNA stability, which is important for the stability of the vaccine following in vivo expression. These findings indicate the feasibility of using the HIV-1 gp120-Nef vaccine as a cost-effective approach for HIV-1 prevention. However, additional in vivo and in vitro studies are imperative to further validate these results.

### 2.20. Vaccine Construct’s Immune Simulations

**Figure 19:**
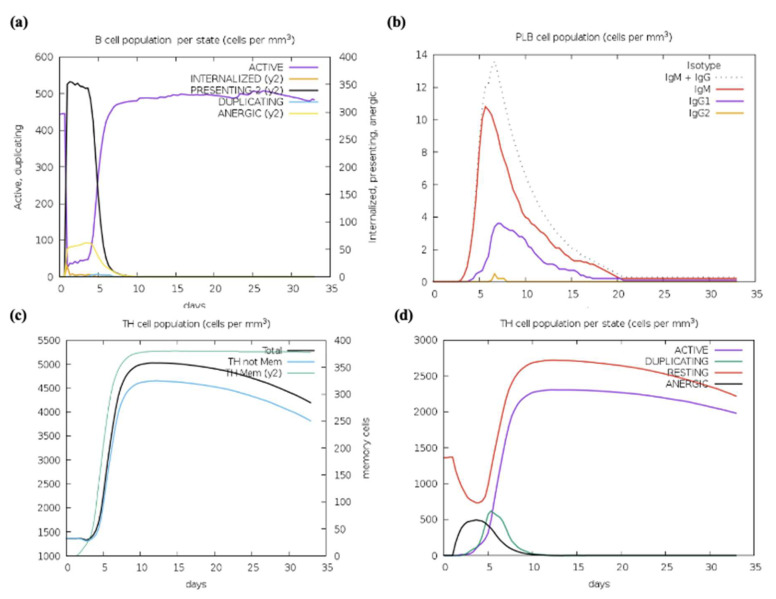

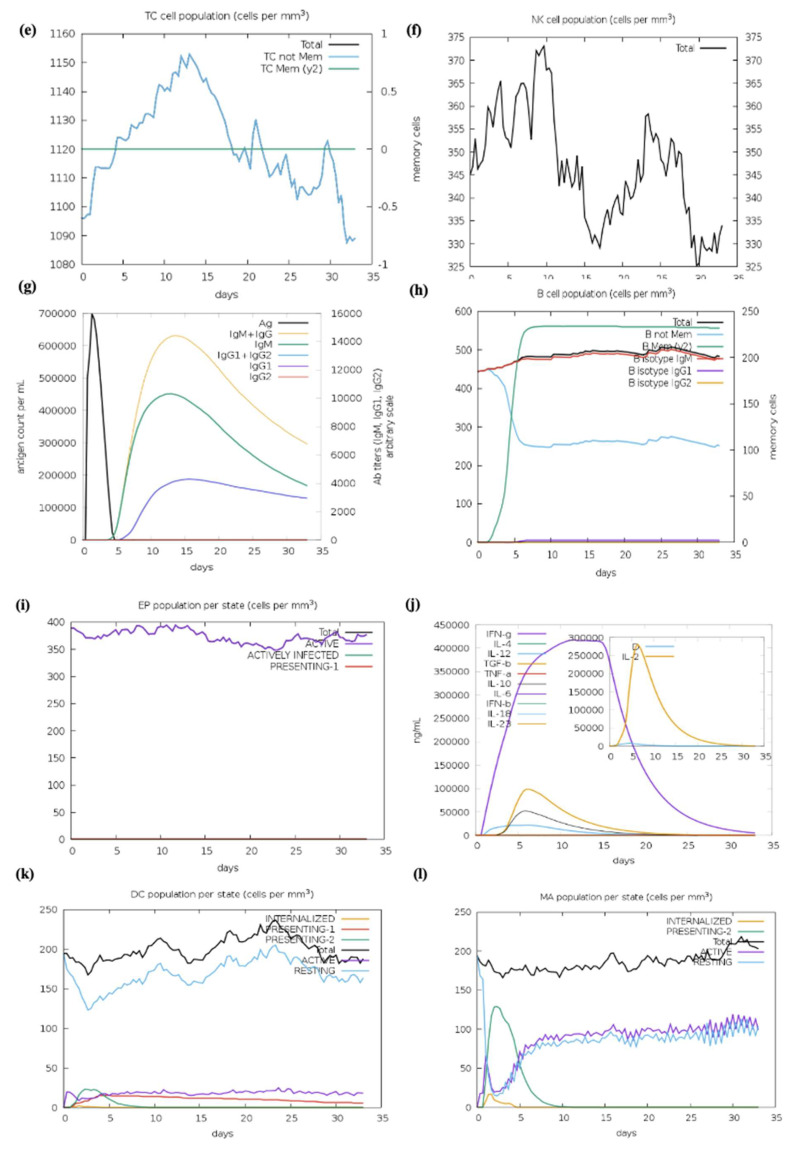
The immune stimulation of the formulated vaccine is represented by C-ImmSimm. (**a**) Colored lines represent the immunoglobulin and immunocomplex reactions to the HIV-1 formulated vaccine inoculations. (**b**–**h**) The steady growth of immune feedback marked by high potency, immunological memory, and effective Ag removal from the host was demonstrated by the elevated numbers of plasma B cells, HTLs, and CTLs. (**i**–**k**) The proliferation of HTL showed the vaccine’s significant adaptive immunity, while the rise in DCs and macrophages showed increased antigen presentation by APCs. (**l**) Production of IFN-γ, IL-23, IL-10, IL-8, and IL-6 is essential for initiating immunological response and defending the body against viruses was demonstrated to be possible with vaccination.

The C-ImmSim program which forecasted the capacity of the chosen epitopes to produce adaptive immune responses was used to evaluate the immunological response elicited by the formulated vaccine. The relationships between the epitopes and their corresponding immune targets were also investigated in this study. The C-ImmSim tool results exposed that the vaccine may produce secondary immune responses that closely resemble actual immunological reactions. The simulation revealed that the HIV-1 gp120-Nef vaccine could elicit strong immune feedback similar to natural immune reactions with stronger primary immune responses observed after each dose (Figure 19a). Moreover, the immune response progressively strengthened with each additional dose, and secondary immune responses showed marked improvement. A strong immune response together with immunological memory and effective Ag removal from the host was demonstrated by a substantial rise in plasma B cells, HTLs, and CTLs (Figure 19b–h). The triggering of HTL further confirmed the vaccine’s capacity to provide significant adaptive immunity, while the simulation also demonstrated an increase in dendritic cells (DCs) and macrophages, which improved Ag presentation by APCs (Figure 19i–k). Furthermore, the vaccine was able to stimulate the production of vital cytokines that are necessary for triggering immunological responses and offering defense against infections, including interleukin-23 (IL-23), IL-10, and IFN-γ (Figure 19l). Significant amounts of APCs, cytokines, and active B and T cells were produced according to the simulation results, indicating that the polyvalent formulated vaccine design will elicit a potent immunogenic response in the host. These results demonstrate that the vaccine promotes strong secondary immune responses after vaccination, improved Ag clearance, and substantial immunological memory.

## 3. Discussion

Computational biology has become a key player in the development of vaccines, particularly in predicting immunogenic peptides that aid in the development of both safe and effective vaccines. By using peptide prediction tools, it is possible to reduce the high costs and minimize adverse side effects typically connected with vaccines derived from attenuated pathogens, whether live or inactive [31]. Even while antiretroviral treatments have made great progress in treating AIDS, an efficient HIV-1 vaccine is still desperately needed to combat this persistent worldwide health emergency [32]. Unfortunately, because they mainly targeted a limited range of HIV genotypes, the majority of prior vaccine candidates were ineffective against the quickly evolving HIV [33]. According to studies on the in silico-designed multiple-epitope EP HIV-1090 vaccine, these vaccines’ capacity to prevent HIV-1 is severely hampered by their inability to provoke robust CTL and HTL responses [34]. Additionally, studies using different multiple-epitope vaccinations on BALB/c mice demonstrated that they are unable to produce broadly neutralizing Abs [35]. The vaccine’s poor effectiveness is also a result of improper cytokine activation and an inability to elicit innate immune responses. Since CTL-mediated immunity is essential for controlling viral infections, a successful HIV-1 vaccine must elicit strong immunological responses, especially by boosting both CTL and HTL responses. Furthermore, HTL-mediated immunity is necessary to support the formation of Abs and to foster a functional CD8^+^ CTL response, both of which contribute to viral control and viral load reduction [36]. When combined with in vivo research, novel immunoinformatic techniques, especially those aimed at identifying multi-functional T cell epitopes, might greatly enhance the design of HIV-1 immunogens [37]. Vaccines based on several epitopes can induce robust HTL and cellular responses. The techniques used in these investigations have opened the door for the development of a vaccine with various epitopes that can stimulate the generation of cytokines like IFN-γ, produce broadly neutralizing Abs, and interact with TLRs to stimulate the optimal innate immune responses. By adding SBP to the vaccine C-terminal, this strategy further improved the immunological response. In silico strategies have become increasingly pivotal in the design of co-epitope vaccines, encouraged by the promising outcomes of numerous studies. Notably, the study demonstrated that a multiepitope construct comprising Nef, Rev, Gp160, and P24 epitopes elicited strong cellular and humoral immune responses in mice. Heterologous prime-boost regimens significantly enhanced IFN-γ, Granzyme B, and IgG2a/IgG2b levels, suggesting their promise as a multiepitope-based HIV-1 vaccine candidate [38]. Similarly, an in silico study identified a truncated p24-Nef fusion protein enriched with conserved CTL epitopes and strong MHC binding, achieving >70% predicted population coverage. These findings support its potential as a promising therapeutic HIV-1 vaccine candidate [39]. A recent study demonstrated that full-length CD40L and IFN-γ, when fused with Nef, enhanced immune responses more effectively than multiepitope constructs. Our results focusing on Nef and gp120 epitopes similarly emphasize the importance of optimized epitope selection, suggesting that combining potent epitopes with strong adjuvant strategies could further improve HIV-1 vaccine design [40]. Similar studies have proposed multiepitope vaccine constructs, where the adjuvant-containing HIV-1b showed enhanced immunogenicity and stability. Our findings on gp120 and Nef epitopes support this strategy, emphasizing the role of conserved, immunogenic epitopes in developing effective HIV-1 vaccines [41]. Similarly, a multiple-epitope vaccine that targets HIV-1 specifically has been developed, which is comparable to what we are currently studying [42].

A vaccine targeting HIV-1 was designed using an immunoinformatic method, paying special emphasis to the Env glycoprotein gp120, which promotes virulence and attachment to the host receptor CD4. Nef also contributes significantly to HIV-1 pathogenesis by promoting viral replication. Since surface glycoproteins constitute the primary site of contact with the host immune system and are widely expressed on viruses, they have long been thought to be excellent candidates for vaccine development. This work used a new and effective computational biology-based technology to develop a co-epitope-based vaccine that targets different strains of HIV-1 by utilizing large amounts of genomic data. The main objective of this effort was to produce a vaccine that could lower the global burden linked to HIV-1, given the effective potential of computational approaches in vaccine development. The consensus sequence served as the foundation for the construction of the 3D structure and anticipated epitopes. While ElliProt was used to provide non-linear predictions, linear epitope predictions for MHC-I and MHC-II binding sites produced a variety of peptide options. Furthermore, the identification of epitopes that may be recognized by Abs was aided by the 3D visualization of gp120 and Nef. A QSAR model was used to estimate IC_50_ values in order to evaluate peptide affinity for MHC; lower IC_50_ values indicate better binding affinity which helps with peptide classification.

Initiating a strong immune response to defend the host from viral infections depends on T cells, particularly HTLs and CTLs. However, compared to vaccines that activate both CTLs and HTLs, those that only target CTL responses have shown decreased efficacy. In light of their unique potential, co-epitope vaccines have a number of advantages over prevalent and single-epitope vaccines. (i) To ensure a varied immune response, a variety of T cell receptors (TCRs) from various MHC class I and II molecules are capable of recognizing comprehensive self and foreign epitopes. (ii) A thorough and well-coordinated defense can be produced by the overlapping CTL, HTL, and B cell epitopes, which can simultaneously elicit humoral and cellular immune feedback. (iii) Adding an adjuvant to the vaccine formulation increases immunogenicity and promotes long-term immunological feedback. (iv) The development of vaccines is made simpler by avoiding issues related to pathogen culture and in vitro Ag expression. To further maximize immunological responses, T cell epitopes that may bind different MHC-I and class II molecules were chosen using the IEDB server. T cell epitopes with a higher binding potential and lower IC_50_ values were selectively targeted by this technique. Antigenic B cell epitopes, on the other hand, interact directly with B cell receptors (BCRs) to start the development of Abs that are confined to particular epitopes. The IEDB server was used to determine if B cell epitopes were linear or continuous. Based on positive results from the initial screening, 122 T cell and B cell epitopes were finalized. The most promising epitopes, which revealed potent antigenic qualities and were devoid of allergens, poisons, and human proteome homologies were found through additional screening rounds. HTL epitopes’ capacity to trigger cytokine reactions, including IFN-γ, IL-4, and IL-10, was also assessed. To enhance the vaccine Ag uptake potential, the SBP was conjugated at the C-terminal. The peptides GTG, GSG, GGTGG, and GGGGS were used to link the epitopes in order to coordinate the vaccine stability and structure. The vaccine immunogenicity, antigenicity, and durability were all enhanced by the conjugation of SBP and the linkers.

It has been demonstrated that longer peptides are more successful than shorter ones at eliciting immunological responses [43]. The ability to develop co-epitope constructions that contain both T- and B cell epitopes, to be efficiently exposed and rectified in order to elicit the intended immune response is still somewhat unknown. Accurately anticipating TAP transport and proteasomal cleavage is essential for improving the effectiveness of multiple-epitope-based vaccines. All of the finalized epitopes including those for CD8^+^ and CD4^+^ T cells are probably attainable for immunological activation during Ag processing and presentation by capable APCs were assessed from the NetCTL 1.2 server. To make sure these epitopes are chosen appropriately for vaccine development, it is crucial to evaluate the immune responses they elicit. An effective, quick, and economical substitute for in vitro and in vivo immunological evaluation is the use of in silico cytokine prediction algorithms, since specific cytokines can be produced by specific residues and motifs within epitopes [44]. IFN-γ is a cytokine that has antiviral, immune-regulatory, and antitumor effects that is essential to both innate and adaptive immunity. Its release plays an important function in lowering the HIV-1 viral load and is essential for the Th1 response [45]. We evaluated the potential of specific HTL epitopes to produce IFN-γ and other cytokines in this investigation. The IFNepitope server was used to anticipate which peptides will attach to MHC-II molecules and produce the secretion of IFN-γ. Based on their anticipated SVM scores, the majority of our chosen HTL epitopes had a strong ability to stimulate IFN-γ and cytokine production. IFN-γ production is intimately associated with HIV-specific T cell immunogenicity and Th1 response activation [46]. According to certain research, IL-10 also has anti-HIV effects by preventing the secretion of inflammatory cytokines [47]. Moreover, studies indicate that T cells that induce IL-10 may aid in lowering HIV replication in expectant mothers [48].

Vaccine immunogens must account for the diversity of HLA tissue types as well as the extensive antigenic variation in HIV-1. In our investigation, we selected peptides containing a range of epitopes with diverse HLA binding specificities to enhance population coverage. MHC-II epitope coverage was 73.35%, whereas MHC-I epitope population coverage was 90.76%. Furthermore, the co-epitope structures exposed a significantly increasing population coverage in areas with high HIV-1 prevalence. The M group of HIV-1 subtypes exhibited a 75% conservation score for the final epitopes. The high conservation across these subtypes decreased the possibility of viral immune evasion, offering broader protection. We used the Vaxigen, AllerTOP v.2.0, and ToxinPred services to evaluate the toxicity, allergenicity, and antigenicity of the final HIV-1 gp120-Nef vaccine design and the anticipated epitopes, respectively. The outcomes verified that the vaccine was non-toxic, non-allergenic, and antigenic. The ProtParam tool from the ExPASy server was used to analyze the physicochemical properties of each predicted epitope as well as the entire vaccine construct. The vaccine exhibited adequate stability with a high pI value of 8.87. The predicted half-life was more than 10 h in a prokaryotic E. coli system and 30 h in mammalian reticulocytes, indicating its potential for stable and efficient production. Additionally, a GRAVY value of −0.646, which is linked to greater water solubility and is also confirmed by predictions from the SolPro server, revealed the vaccine’s hydrophilic character.

To confirm the protein closely resembled its original structure, the formulated vaccine 3D structure was first modeled using the RoseTTAFold online server and then improved using the GalaxyRefine server. Our results showed that the quality and accuracy of the projected 3D structures increased when the vaccine build was enhanced. When the 3D and improved structure of the formulated vaccine was evaluated, the results were further verified using the Ramachandran plot and the Z-score analysis. This research supported the vaccine’s potential efficacy by confirming that it assumed a structurally appropriate conformation. The vaccine Z-score was −6.05, which is within the permissible range for protein crystal structures confirmed by experiment. Crucially, the Ramachandran plot favorable areas had the largest concentration of amino acids [49]. Furthermore, we conducted protein-protein docking studies between TLRs and the projected constructions. Through pathogen detection and adaptive immune response, TLRs are essential for innate immune system activation [50]. In particular, TLR3 is in charge of activating DCs in response to HIV-1, whereas TLR2 and TLR4 identify viral structural proteins and start the induction of inflammatory cytokines [51].

According to the methodology section, we used an in silico experiment to determine the interaction between the vaccine construct and a number of TLRs in our investigation. Low energy values and a high binding potential between the vaccine construct and the TLRs were found in the results. Different patterns of contacts between interchain residues in the TLR-vaccine complexes were revealed by contact map analysis. Interestingly, there were more interchain interactions between different protein domains in the TLR2-vaccine complex. Three distinct sites on the contact map exposed interchain interactions between various domains in the TLR3 and TLR5 vaccine complexes. In the TLR4-vaccine complex, fewer interchain interactions were seen, indicating a weaker TLR-vaccine chain binding. Whereas the TLR8-vaccine complex exposed contact between several vaccine chain residues and the TLR chain, the TLR7-vaccine complex only showed interactions between residues that were another. These outcomes propose that the developed vaccine designs may activate downstream signaling pathways and engage TLRs, resulting in the generation of pro-inflammatory cytokines that may take part in the fight against HIV-1 infection. Both humoral and cellular immune feedback should be elicited by a strong HIV-1 therapeutic vaccine. Inflammatory gene transcription is triggered by a TRIF-dependent signaling cascade that is initiated by TLR-3 activation [52]. This activation promotes the development of DCs into potent immunostimulatory cells capable of cross-priming T cells. TLR-3 signaling is required for DC activation in HIV-1 infection [53]. Conversely, TLR-4 stimulation causes IL-6 to be produced, which has the potential to reactivate latent HIV-1 [54]. TLR-10 has also been connected to increased HIV-1 infection [55].

To evaluate the vaccine-receptor complex’s equilibrium and binding efficiency, MD simulations were used. The pathway by which the vaccine and TLRs interact over time was shown by these simulations. The findings indicated a high binding affinity since the vaccine design efficiently occupied the TLRs with little energy. Furthermore, iMODS normal-mode analysis exposed that the vaccine complex had high eigenvalues, which suggested a lower degree of deformability. This implies that the vaccine’s structure is comparatively inflexible and impervious to fluctuation in shape. This conclusion was corroborated by the deformability graph, which showed that the vaccine is probably going to keep its stability and structural integrity. Furthermore, useful details regarding the stability and conformational changes in the physiological system were provided by atomistic simulations. By analyzing multiple indicators derived from the MD simulation trajectory data, we were able to examine the stability and conformational changes in the vaccine–receptor complex in greater detail [56]. Our comprehension of the vaccine construct’s dynamic behavior and structural characteristics in combination with TLRs is improved by these simulations.

We optimized the vaccine mRNA using the JCAT tool and the *E. coli* K-12 strain as the cell culture system. This platform was used to evaluate the translation efficiency of the final vaccine design. The outcomes showed a CAI of 0.95 and a GC content of 54%, both of which are considered optimal as a CAI score greater than 0.60 and a GC percentage of 30% to 70% are generally regarded as desirable. The pET plasmid’s codon optimization process involved the use of *Nco*I and *Xho*I restriction enzymes to cleave the N- and C-termini, respectively. The insertion of a HisTag into the plasmid enables efficient post-translational purification of the vaccine. Moreover, the secondary structure of the final vaccine construct was evaluated using the RNAfold software (version 2.1.0), which revealed a minimum free energy score of −806.89 kcal/mol, suggesting improved stability for the vaccine in the body. For the vaccine to work effectively, its stability is essential. However, promising results were obtained from in silico evaluation of the host immune feedback to the developed co-epitope vaccine. Both B and T cell activation were anticipated by the C-ImmSim algorithm, resulting in immunological memory that lasts a lifetime. Strong protection against the virus is indicated by the induction of IgG1 and IgG2 Abs, which suggests the activation of Th1 and Th2 responses against HIV-1 Ags. Furthermore, the ICM server data demonstrated that a rise in CTL levels was correlated with an increase in Th1 cell numbers. The vaccine design which contains immunogenic B and T cell epitopes from gp120-Nef, appears to have significant potential for developing a co-epitope vaccine against HIV-1 according to these results taken together. However, additional in vitro and in vivo research is necessary to completely assess our vaccine potential.

## 4. Materials and Methods

### 4.1. Exploration of Protein Sequences

The GenBank database server was used for the sequence retrieval for the identification of gp120 and Nef proteins associated with HIV-1. The STRAP program was utilized to conduct multiple sequence alignment to analyze different variable and conserved regions within the gp120 and Nef proteins. A consensus sequence was then generated using the MUSCLE server. Table 1 mentions all the computational tools used in this study.

### 4.2. Epitope Prediction for B Cells

Two computational tools, such as BepiPred-2.0 and the BepiPred Linear Epitope Prediction tool accessible through the IEDB resource, were used in this investigation to assess linear B cell epitopes within gp120 and Nef proteins. During analysis, both tools were utilized with their default threshold values [57,58]. The random forest machine learning technique used by BepiPred-2.0 was trained on structural data from known Ab-antigen complexes. This technique combines sequence-based prediction improved by 3D structural information with an extensive dataset of linear epitopes from IEDB.

To increase confidence in the predicted epitopes, the results were further endorsed by employing the iBCE-EL platform. iBCE-EL is an online tool that identifies linear B cell epitopes by combining various input features such as dipeptide composition, physicochemical properties, and machine learning algorithms, including extremely randomized trees and gradient boosting methods. It generates both classification and probability scores for each peptide sequence submitted [59].

B cell epitopes are in large numbers and belong to the discontinuous category, which reveals that amino acid residues in these epitopes are far distant in their linear sequence yet physically nearby in the protein’s 3D structure. The ElliPro program was assessed to anticipate conformational type epitopes in the final vaccine design 3D model. The ElliPro analyzes the protein’s structural geometry and has demonstrated high accuracy with an AUC score of 0.732, ranking it among the most effective tools for identifying conformational Ab-binding sites.

### 4.3. CTL Epitope Prediction

The IEBD analysis library assembled the NN-align platform was used to analyze the epitopes to find MHC-I-binding alleles, including both common and uncommon variants. Peptide lengths ranging from 8 to 12 residues and an IC_50_ threshold value of less than 100 were among the characteristics used in the analysis for MHC-I-binding potential. IC_50_ values in the peptides found less than 50 nM were regarded as having strong binding affinity, while moderate affinity was considered among those below 500 nM, and weak affinity was considered among those below 5000 nM. Consequently, a stronger binding affinity is indicated by a smaller IC_50_. Furthermore, several characteristics of the selected epitopes, such as processing scores, proteasomal cleavage locations, TAP values, and MHC-I-binding tendency, were predicted using the IEDB server. The NN-align algorithm was employed for the determination of all these parameters.

### 4.4. Evaluation of MHC-I Immunogenicity 

The IEDB MHC-I immunogenicity prediction tool was used to assess MHC peptides that might be immunogenic within the infected host cell. The server’s default parameters were employed to evaluate the chosen epitopes. In order to proceed with a further experimental plan, only highly immunogenic peptides were selected from the predicted epitope list.

### 4.5. HTL Epitope

HTLs are key players in the immune feedback function by recognizing the Ags and modulating the immune system to activate B cells as well as CTLs. MHC-II T cell epitopes were evaluated using the Immune Epitope Database website, and percentile rank and MHC binding affinity parameters were utilized to predict epitopes. The IEDB-recommended conjunctional approach used techniques like NetMHCIIpan, Sturniolo, CombLib, and SMM-align to identify HTL epitopes [59]. The selection of final epitopes was considered on their IC_50_ score lower than 500 nM, their ability to elicit IFN-γ release, their propensity to show emerging characteristics, and their binding scores (lower scores indicate stronger binding affinity).

### 4.6. Selection of Promising Epitopes

Based on knowledge gained from earlier studies, the thresholds for epitope selection were established. Finding a compromise between assuring specificity and optimizing accuracy to locate immunogenic epitopes was the aim. The selection of the final epitopes was based on different numbers of immunogenic tests as well as their physicochemical properties. This dataset is primarily focused on HIV-1 since it offers a thorough compilation of immunological and non-immunogenic epitopes after a meta-analysis of studies with an emphasis on MHC-I epitopes.

### 4.7. Proteasomal Cleavage/TAP Transport

The process of predicting protease cleavage, as well as transporter-associated antigen processing (TAP), was performed via the NetCTL 1.2 method. Through a series of algorithms, the computer evaluates the efficacy of MHC-I binding, TAP transport, and proteasomal C-terminal cleavage. The predictions were performed using the default parameters with the TAP transport ability set at 0.05 in weight and the C-terminal cleavage assigned a weight of 0.15.

### 4.8. Prediction of IFN-γ Cytokine Inducer

Epitopes with the capability of eliciting the cytokine IFN-γ were predicted via the online server. A dominant Th1 immune response cytokine for antiviral activity in both innate and adaptive immune systems is IFN-γ. Amongst others, this method predicts MHC-II binding peptides which can boost the production of IFN-γ by CD4^+^ T cells. The server parameters were then changed to select the SVM-based method, so that IFN-γ production is favored over the production of other cytokines.

### 4.9. Population Coverage Assessment

The coverage of particular epitopes in the population was ascertained using the population coverage tool available on the IEDB website. The world’s population was evaluated using this tool. The optimal epitopes for different human leukocyte antigen (HLA) bindings can be chosen with the use of population coverage estimation. In order to offset the burden of MHC limitation in T cell responses, different ethnic groups may carry epitopes with higher HLA binding affinities; hence, those groups may have different frequencies. In this study, the HLA binding alleles for MHC-I and MHC-II were assessed for HLA binding using the gp120 with Nef proteins. Additionally, the peptide sequences from several HIV-1 subtypes in group M were examined using the IEDB epitope conservation analysis program in order to find conserved cross-reactive epitopes.

### 4.10. Antigenicity, Allergenicity, and Solubility Prediction

The antigenicity of the finished vaccine construct and its components was evaluated using the AntigenPro and VaxiJen 2.0 servers. VaxiJen depends on the primary amino acid characteristics and the auto cross-covariance (ACC) transformation, which converts protein sequences to fixed vectors [60]. AntigenPro, in contrast, is a predictor of protein antigenicity pathogen-independent sequence [61]. To evaluate the allergenicity of the vaccine and its constituent parts, AllergenFP 1.0 and AllerTOP 2.0 were applied. AllergenFP classifies allergens using a binary classifier and blends non-allergic proteins to the set delimited by five E-descriptors. The ACC transformation translates these descriptors to fixed vectors [62]. E-descriptors and the ACC transformation are also utilized by AllerTOP. The Protein-Sol and SolPro tools assessed the solubility of the components of the vaccine. SolPro uses support vector machines (SVM) to predict protein solubility with an accuracy of approximately 74% based on tenfold cross-validation. Protein-Sol, however, predicts *E. coli* soluble proteins, drawing information from a system devoid of cells [63].

### 4.11. Toxicity and Physicochemical Characteristics Assessment

The final vaccine construct toxicity and its ingredients were analyzed using the ToxinPred program. ToxinPred uses an SVM model to categorize proteins according to their characteristics as either hazardous or non-toxic [64]. Furthermore, the vaccine and its constituent physicochemical attributes were assessed with the ExPASy ProtParam tool, which analyzes the charge, hydropathicity, half-life, instability index, theoretical isoelectric point (pI), and molecular weight [65,66].

### 4.12. Epitope Hydropathy Analysis

As HIV-1 vaccines need to be displayed on the protein surface for immune response activation, all epitopes must be hydrophilic. Evaluating the hydrophobicity of epitopes required for computing the GRAVY (Grand Average of hydropathy) score, which was performed using the ProtParam service, was one of the steps. Each protein’s total hydropathy values are added up, and the result is divided by the total number of amino acid residues to determine the GRAVY score. A hydrophobic protein is indicated by a positive GRAVY score, whereas the existence of hydrophilic areas is shown by a negative score.

### 4.13. Cluster Analysis of MHC-Restricted Alleles

We developed a set of epitopes for presentation on MHC class I/II molecules using the online IEDB. We also used the MHCcluster v2.0 server to further validate these predictions, which revealed more proof. This site uses a static heat map to illustrate the connection between peptides and HLA function [67].

### 4.14. Design of Co-Epitope Subunit Vaccine

The most promising epitopes were carefully analyzed, which included enhanced antigenic potential, non-toxicity, non-allergenicity, no homology to the human proteome, and conservation among specific strains. HTL epitopes that could elicit cytokine responses were selected for vaccine formulation after their capacity to trigger cytokine production was assessed. Potential epitopes were preferred for the construction of a co-epitope final vaccine design that targets HIV-1 after fulfilling the requirements and passing the aforementioned analysis. The final potential epitopes from B cells, HTL, and CTL included in the vaccine design and conjugated with the SBP sequence using various linkers at the C-terminal. Phage display screening was used in our lab to identify the SBP. A human liver cDNA expression library was screened against HBsAg to identify this 344-amino-acid protein. A GGGGS linker connected the SBP peptide to the epitopes, and its conjugation boosted the immunological response that the vaccine elicited.

### 4.15. Evaluation of the Vaccine Structure Physicochemical Characteristics

Assuring high antigenicity is essential to vaccine production because it guarantees that the vaccine will be recognized by the host immune system, following immune cell stimulation and subsequent immunological responses [68]. Allergenicity testing is necessary to avoid allergic reactions in the host. Additionally, a deeper analysis of the vaccine physicochemical characteristics is important to guarantee its efficacy and safety. The VaxiJen v2.0 tool, with a highly reliable threshold of 0.4, was used for antigenicity evaluation of the final vaccine sequence. AlgPred and AllerTop v2.0 were also used to assess the vaccine’s allergenicity. AlgPred compares frequent epitopes in protein regions using different approaches to predict antigenic and possible allergenic potential. After an allergenicity screen was performed using the MEME/MAST motif prediction tool from AllerTop v2.0, physicochemical properties like pI value, half-life, GRAVY index, etc., were predicted using the ProtParam tool. Conversely, the SCRATCH protein predictors SOLpro tool was used to forecast the vaccine construct solubility while maintaining the default parameters. The Protein-Sol tool was used to cross-verify the results. Solubility is crucial to ensuring that the vaccine remains soluble when administered to the host. If a vaccine collects into insoluble particles, it may lose its effectiveness. Protein-Sol predicts solubility using a fast sequence-based algorithm, while SolPro uses an SVM-based method. These resources offer trustworthy approximations of protein sequence solubility.

### 4.16. Prediction of Vaccine Secondary and Tertiary Structures

The structural conformation of a protein largely determines its function; therefore, both secondary and tertiary structures should be carefully considered in vaccine design. Following antigenicity and allergenicity testing, the online PRISPRED application was sectionalized for secondary structure analysis of the vaccine while maintaining all default values. Secondary structural features, such as folds, helices, transmembrane topology, and domain recognition, are analyzed using PRISPRED [68]. To provide an additional layer of explanation for these findings, additional 2D structural studies were performed utilizing the SOPMA and Phyre2 servers for comparison and validation. We constructed a 3D model for the vaccine using the RoseTTAFold (BOINC version 7.6.22) program. RoseTTAFold combines a protein sequence pattern, amino acid interactions between proteins, and possible 3D structures into a neural network-based “three-track” algorithm. This thorough approach makes it possible to comprehend a protein’s folded structure and chemical makeup from all angles. RoseTTAFold has shown remarkable accuracy levels that are on par with AlphaFold from DeepMind. The technology provides a comprehensive understanding of a protein’s structure by allowing information to move between one-, two-, and 3D levels [69].

### 4.17. Validation and Improvement of the 3D Vaccine Structure

When designing vaccines, computer-based techniques are essential, especially when there is a lack of experimental data. In biomedical applications, the development of 3D models is a crucial first step, but it is not always enough to guarantee accuracy; experimental data is necessary for confirmation. It is feasible to enhance the quality of initially created models by using 3D structure refinement, which fixes local mistakes while maintaining the important structure of the vaccine. The GalaxyRefine tool was used to enhance the vaccine’s 3D structure. This server enhances structural quality through dynamic simulation and refinement using a CASP10-tested technique [70]. It is still difficult to refine 3D vaccine models consistently and precisely, particularly when working with high-resolution data. The PROCHECK server was used to develop Ramachandran plots in order to verify the vaccine design’s accuracy [71]. With the van der Waals radius of side chains taken into account, these figures evaluate the permitted and prohibited dihedral angles (psi and phi) of the amino acid organizations. Furthermore, the ProSA-web application was utilized, which computes a Z-score for protein structure validation using statistical techniques. We could evaluate the consistency and quality of the vaccine’s design by comparing the Z-score, which represents the standard of the protein composition and with the Z-scores from the PDB database for experimentally determined protein structures [72].

### 4.18. Vaccine Disulfide Engineering Evaluation

Vaccine design undoubtedly encompasses the enhancement of the stability of vaccines by way of numerous molecular interactions. Disulphide by Design 2 v12.2 was used to analyze disulfide regions in the vaccine construct. This tool works to locate expected sites within a protein structure where disulfide bonds can be formed. Based on the 5th Cbeta-Cbeta distance, the server computationally predicts protein structures to precisely determine the χ3 torsion angle and uses this as a geometric framework constructed from naturally occurring disulfide bonds [73].

### 4.19. Vaccine Construct Docking with TLRs

Drug design and the study of protein–protein interactions both strongly rely on docking. It entails using protein-protein docking to combine distinct protein structures in order to anticipate the structure of a complex. The final vaccine structure was docked with multiple TLRs in this study, including TLR-2, 3, 4, 5, and 8 (PDB IDs: 2Z7X, 1ZIW, 3FXI, 3J0A, and 3W3G), to perform protein-protein docking. PyMOL v2.3.4 was used to increase the 3D architectures of the vaccine complex and TLRs for energy minimization. Several online docking technologies were employed to evaluate vaccine-receptor interactions in order to enhance prediction quality. Before docking, water molecules were eliminated from the PDB structures of HIV-1 gp120-Nef, TLRs, and the vaccine. During the docking study, the Chimera v1.13.1 tool was employed to assess the 3D configuration of the formulated vaccine structure. The HADDOCK server was used for the assessment of bonding areas between the vaccine construct and TLR2. The HADDOCK study chose the best-ranked complexes based on the lowest mean RMSD and the lowest intermolecular binding energy, which represent the complete HIV-1 gp120-Nef-TLR interaction.

ClusPro 2.0 was used to sort the clusters of docked complexes in the second docking phase with an emphasis on the lowest and center energy values [74]. The default docking parameters were used for the MM-GBSA (Molecular Mechanics/Generalized Born Surface Area) study. MD simulation was employed to predict the reciprocity between the TLRs and the vaccine construct in greater detail following successful docking. The docked complexes were visualized using the Discovery Studio Visualizer. Furthermore, the protein–protein interactions within the complexes were examined using PDBsum [75]. The LigPlot^+^ v.2.2.4 software was used to evaluate H-band formation. The CB-Dock2 web server was utilized to identify potential ligand-binding pockets in the designed protein structure. The server detected and ranked cavities based on their volume and spatial properties. CB-Dock2 provided detailed information on the volume, coordinates (x, y, z), and size of each pocket, facilitating the selection of suitable binding sites for subsequent molecular docking studies.

### 4.20. Vaccine-Receptor Complex MD Simulation

MD simulations with iMOD were used to explore transition paths between two homologous structures. The server performs normal mode analysis (NMA) to calculate essential protein stability characteristics through its internal computation process. Protein stability can be assessed through key components, which include the elastic network model together with the covariance matrix and eigenvalues, as well as B-factor values and the main-chain deformability plot. This inquiry provides a better understanding of protein stability together with its structural dynamics [76].

### 4.21. Vaccine Construct Codon Optimization and In Silico Cloning

For the peptide vaccine transcription to produce an appropriate DNA sequence, the targeted protein was reverse-translated. The target protein’s efficient expression in the selected organism was subsequently ensured by optimizing the DNA sequence for codon use. The Java Codon Adaptation Tool conducted the process of codon adaptation for the recommended vaccination protein through its service [77]. The selected host organism for this study consists of the *E. coli* K-12 bacterial strain. During the adaptation step, we excluded prokaryotic ribosome-binding regions as well as rho-independent transcription terminators and *Nco*I and *Xho*I restriction enzyme cleavage regions. We employed the NcoI and XhoI restriction regions to integrate the optimized DNA sequence into the pET-28a vector conjugated with HisTag. The HisTag functioned to facilitate protein solubilization and purification during the affinity-based process [77,78].

### 4.22. Vaccine Constructs Immune Simulation

The forecasted immune response to the computationally designed vaccine construct was assessed through the C-ImmSim server. The server combines agent-based simulation with machine learning algorithms to generate immune response predictions through the position-specific scoring matrix (PSSM). The simulation ran through 1050 steps to replicate the interval of four weeks between vaccine doses. The simulation process treated both injections through a virtual eight-hour period to match the real-time sequence of events. The analysis preserved all default parameter values throughout its execution.

## 5. Conclusions

The discovery of immunogenic epitopes which are necessary for the development of effective vaccines is greatly aided by bioinformatics. In this investigation, the HIV-1 gp120 and Nef structural proteins were the targets of a co-epitope vaccine. Potential linear B cell, HTL, and CTL epitopes were combined to formulate the vaccine. First, the chosen epitopes were evaluated for allergenicity, toxicity, and antigenicity. Subsequently, these epitopes were conjugated to SBP and several linkers. For the selected parameters, the vaccine’s physicochemical study and 3D structural validation produced encouraging outcomes. Molecular docking analysis revealed strong binding affinities of the vaccine construct with receptors such as TLR3, TLR4, TLR5, and TLR7. Additionally, immune simulation and MD simulations provided favorable results. Nevertheless, further research is required to evaluate the potential of the vaccine in triggering anti-HIV-1 immune responses, including Ab production and cytokine activation in animal models with the ultimate goal of reducing HIV-1 infection.

## Figures and Tables

**Table 1:** Several computational analysis tools were used in the vaccine formulation.

Tool Name	Prediction	Tool Source
EMBL-EBI	Consensus sequence	https://www.ebi.ac.uk/jdispatcher/ (accessed on 4 March 2024)
IEDB	B cell analysis	http://tools.iedb.org/bcell/ (accessed on 4 March 2024)
iBCE-EL	B cell analysis	http://thegleelab.org/iBCE-EL/ (accessed on 4 March 2024)
BepiPred-2.0	B cell analysis	http://www.cbs.dtu.dk/services/BepiPred/ (accessed on 4 March 2024)
ElliPro	B cell analysis	http://tools.iedb.org/ellipro/ (accessed on 4 March 2024)
IEDB	HTL analysis	http://tools.iedb.org/bcell/ (accessed on 4 March 2024)
IEDB	Immunogenicity	http://tools.iedb.org/mhcii/ (accessed on 4 March 2024)
IEDB	CTL analysis	http://tools.iedb.org/mhci/ (accessed on 4 March 2024)
AntigenPro	Antigenic potential	http://scratch.proteomics.ics.uci.edu/ (accessed on 4 March 2024)
VaxiJen 2.0	Antigenic potential	https://www.ddg-pharmfac.net/vaxijen/VaxiJen/VaxiJen.html (accessed on 4 March 2024)
ToxinPred	Epitope toxicity	http://crdd.osdd.net/raghava/toxinpred/ (accessed on 4 March 2024)
AllerTOP 2.0	Allergenicity	https://www.ddg-pharmfac.net/allertop_test/ (accessed on 4 March 2024)
AllergenFP 1.0	Allergenicity	https://ddg-pharmfac.net/AllergenFP/ (accessed on 4 March 2024)
ExPASy	Physicochemical characteristics	https://web.expasy.org/protparam/ (accessed on 5 March 2024)
Protein-sol	Solubility	https://protein-sol.manchester.ac.uk/ (accessed on 5 March 2024)
SolPro	Solubility	http://scratch.proteomics.ics.uci.edu/ (accessed on 5 March 2024)
Phyre2	Secondary structure	https://www.sbg.bio.ic.ac.uk/~phyre2/html/page.cgi?id=index (accessed on 5 March 2024)
PRISPRED	Secondary structure	http://bioinf.cs.ucl.ac.uk/psipred/ (accessed on 5 March 2024)
SPOMA	Secondary structure	https://prabi.ibcp.fr/htm/site/web/app.php/home (accessed on 5 March 2024)
MHCcluster v2.0	MHC cluster prediction	https://services.healthtech.dtu.dk/services/MHCcluster-2.0/ (accessed on 5 March 2024)
RoseTTAFold	Tertiary structure	https://robetta.bakerlab.org/ (accessed on 5 March 2024)
PROCHECK	Ramachandran plot	https://saves.mbi.ucla.edu/ (accessed on 6 March 2024)
GalaxyWEB	Structure improvement	https://galaxy.seoklab.org/ (accessed on 6 March 2024)
ProSA-web	Z score	https://prosa.services.came.sbg.ac.at/prosa.php (accessed on 6 March 2024)
Design 2 v12.2	Disulfide engineering	http://cptweb.cpt.wayne.edu/DbD2/ (accessed on 6 March 2024)
Chimera V 1.13.1	Protein analysis	https://www.cgl.ucsf.edu/chimera/olddownload.html (accessed on 6 March 2024)
ClusPro 2.0	Protein docking	https://cluspro.bu.edu/login.php (accessed on 6 March 2024)
HADDOCK	Protein docking	https://rascar.science.uu.nl/ (accessed on 6 March 2024)
PDBsum	Protein–protein interaction	https://www.ebi.ac.uk/thornton-srv/databases/pdbsum/Generate.html (accessed on 6 March 2024)
LigPlot^+^	Protein interaction	https://www.ebi.ac.uk/thornton-srv/software/LigPlus/ (accessed on 6 March 2024)
iMOD	Molecular dynamics simulation	http://imods.chaconlab.org (accessed on 6 March 2024)
SnapGene	Cloning	https://www.snapgene.com/ (accessed on 6 March 2024)
C-ImmSim	Immune simulation	https://kraken.iac.rm.cnr.it/C-IMMSIM/index.php (accessed on 6 March 2024)

**Table 2:** B cell linear epitopes analyzed from gp120 and Nef proteins.

Length	Epitope	Protein	Antigenicity	Allergenicity	Toxicity	GRAVY
31–37	KAYDTEV	Gp120	0.3604	No	No	−0.986
51–56	PNPQEV	Gp120	1.3029	Yes	No	−1.583
104–124	EVKNDTNTNPPPGRMIMEKSD	Gp120	0.1711	No	No	−1.284
136–147	IRGKVQKEYAFF	Gp120	0.6248	No	No	−0.307
173–181	TQACPKVSF	Gp120	1.1348	No	No	1.089
271–281	NNNTRKRPRIIQ	Gp120	0.9724	No	No	−3.0
333–342	GGSSGGDPEI	Gp120	0.486	No	No	−0.99
368–384	FNSTWSTEGSNNTEGSD	Gp120	0.6401	Yes	No	−1.482
404–410	KAMYAPP	Gp120	0.6789	No	No	−0.414
461–480	VKIEPLGVAPTKAKRRVVQR	Gp120	1.1063	No	No	−0.39
4–13	KWSKSSVIGW	Nef	0.8573	Yes	No	−0.37
90–107	FLKEKGGLEGLIHSQRRQ	Nef	0.4644	Yes	No	−0.961
119–136	GYFPDWQNYTPGPGVRYP	Nef	0.5657	No	No	−1.172
149–162	EPDKIEEANKGENT	Nef	0.6293	Yes	No	−2.05
171–178	HGMDDPER	Nef	−0.1358	No	No	−2.288
105–202	ARELHPEY	Nef	1.4148	No	No	−1.5

Different IEDB servers were used to identify the B cell epitopes. These epitopes were chosen according to their antigenic qualities, negative GRAVY values, non-toxicity, and non-allergenicity. To make sure they were appropriate, all epitopes were also evaluated for surface accessibility and flexibility. Epitopes are categorized by their parent protein (gp120 or Nef) and given in ascending order of sequence length.

**Table 3:** IEDB database server-predicted CTL gp120 epitopes.

Length	Peptide	Antigenicity	Allergenicity	Toxicity	Gravy	IC_50_	PCC	TAP
31–40	KAYDTEVHNV	0.4615	No	No	−0.94	75	0.2853	−0.9710
32–40	AYDTEVHNV	0.4509	No	No	−0.611	89	0.9323	0.5450
47–56	VPTDPNPQEV	0.8561	No	No	−1.11	77	0.4364	−1.8460
57–65	VLVNVTENF	0.4762	No	No	0.889	50	0.9419	2.6920
81–90	SLWDQSLKPC	0.2043	No	No	−0.49	54	0.8349	0.3470
90–100	QAHCNISRAKW	0.2319	No	No	−0.882	36	0.5544	−0.4420
105–115	KQIASKLREQF	0.2997	No	No	−0.973	11	0.0387	−1.3160
107–115	IASKLREQF	1.0623	No	No	−0.367	3	0.5895	2.6040
132–140	ISTSIRGKV	0.6048	No	No	0.233	69	0.7683	0.3400
139–148	KVQKEYAFFY	0.6481	No	No	−0.58	46	0.5240	2.7460
140–148	KVQKEYAFF	0.772	No	No	−0.5	14	0.5514	2.7460
153–162	FNSTWFNSTW	0.4342	No	No	−0.62	47	0.2752	−0.9590
171–179	VITQACPKV	0.3301	No	No	0.3301	98	0.9082	0.4670
178–186	KVSFEPIPI	2.4609	No	No	0.511	38	0.8604	0.7740
179–188	VSFEPIPIHY	2.286	No	No	0.4	74.16	0.5901	−0.3230
179–189	KVSFEPIPIHY	2.2045	No	No	0.009	28	0.8193	0.7740
180–188	SFEPIPIHY	2.1496	No	No	−0.022	74.16	0.9781	2.9990
180–189	VSFEPIPIHY	2.2860	No	No	0.4	67	0.5901	−0.3230
188–196	YCAPAGFAI	1.3004	No	No	1.322	23	0.3413	0.5500
251–259	NAKTIIVQL	0.0323	No	No	0.8	81	0.9752	1.0400
267–275	CTRPNNNTR	0.7904	No	No	−2.222	66	0.8586	1.4690
305–313	SRAKWNNTL	0.5984	No	No	−1.356	71	0.9742	1.2290
359–367	TQLFNSTWF	−0.1535	No	No	−0.078	12	0.8403	2.6050
351–383	DTITLPCRI	0.7402	No	No	0.478	29	0.5292	0.2640
385–393	ITLPCRIKQ	1.3668	No	No	0.122	51	0.2214	−0.0880
398–406	WQKVGKAMY	0.6072	No	No	−0.667	31	0.9513	2.9690
404–414	AMYAPPISGQI	0.4757	No	No	0.482	81	0.5113	−0.9450
406–414	YAPPISGQI	0.6513	No	No	0.178	22	0.9569	0.5500
468–476	APTKAKRRV	0.9750	No	No	−1.256	66	0.9213	0.1160
469–477	PTKAKRRVV	0.6876	No	No	−0.989	17	0.4216	−0.1910

The IEDB server was used to anticipate CLT epitopes. The selection of gp120 epitopes was determined on their antigenicity rate, IC50 value, % rank, and binding potential to MHC-I HLAs (nM). The TAP transport and proteasomal C-terminal cleavage potential of the chosen epitopes were further assessed. For each of the chosen epitopes, the level of conservation was considered. Epitopes in the table are arranged according to their starting position in the protein sequence, from the N-terminal to the C-terminal.

**Table 4:** IEDB database server- predicted CTL Nef epitopes.

Length	Peptide	Antigenicity	Allergenicity	Toxicity	Gravy	IC_50_	PCC	TAP
4–14	KWSKSSVIGWP	0.5857	No	No	−0.482	21	0.1115	−1.0480
8–16	SSVIGWPTV	0.3056	No	No	0.856	34	0.9587	0.4840
60–68	AQEEEEVGF	1.4947	No	No	−1.011	78	0.5785	2.6080
65–74	EVGFPVTPQV	1.0911	No	No	0.41	68	0.3469	−0.3360
66–74	VGFPVTPQV	−0.1141	No	No	0.844	78	0.9646	0.2200
66–74	VGFPVTPQV	0.9787	No	No	0.844	77	0.9646	0.2200
76–84	MTYKAAVDL	0.4955	No	No	0.456	86	0.9558	1.1650
78–87	MTYKAAVDL	0.4955	No	No	0.456	12	0.9558	1.1650
82–90	KAAVDLSHF	0.5513	No	No	0.333	97	0.8515	2.7090
83–91	AAVDLSHFL	0.5768	No	No	1.189	23	0.9579	1.2270
90–100	FLKEKGGLEGL	0.2584	No	No	−0.164	43	0.0931	−1.6450
103–112	SQRRQDILDL	1.6028	No	No	−1.17	19	0.0558	−1.6400
115–124	YHTQGYFPDW	0.6024	No	No	−1.36	87	0.1841	−1.8660
119–127	GYFPDWQNY	0.6274	No	No	−1.467	39	0.9755	3.1200
125–135	QNYTPGPGVRY	0.9195	No	No	−1.327	86	0.6341	0.3600
126–135	NYTPGPGVRY	1.0343	No	No	−1.11	91	0.9185	1.7480
133–143	VRYPLTFGWCY	0.8815	No	No	0.236	21	0.9767	1.3380
137–145	LTFGWCYKL	1.3311	No	No	0.633	12.85	0.9735	1.0470
162–170	TSLLHPVSL	0.6512	No	No	0.944	29	0.9625	0.9900
172–180	GMDDPEREV	0.0303	No	No	−1.6	5.27	0.9455	0.0790
180–189	VLEWRFDSRL	1.3121	No	No	−0.31	18	0.6160	1.4200
182–191	EWRFDSRLAF	1.6334	No	No	−0.65	32	0.2322	−0.4460
192–202	HHVARELHPEY	0.6975	No	No	−1.291	24.72	0.0733	0.0970
194–202	VARELHPEY	1.1079	No	No	−0.867	14.86	0.9752	3.0860
195–202	ARELHPEY	1.4148	No	No	−1.5	56	0.9341	1.1250
195–203	ARELHPEYF	1.0785	No	No	−1.022	10	0.8923	2.7390
194–202	VARELHPEY	1.1079	No	No	−0.867	17	0.9752	3.0860

The IEDB server was used to anticipate CLT epitopes. Nef epitopes were chosen according to their antigenicity rate, IC_50_ value, % rank, and binding potential to MHC-I HLAs (nM). The TAP transport and proteasomal C-terminal cleavage potential of the chosen epitopes were further assessed. For each of the chosen epitopes, the level of conservation was considered. Epitopes in the table are arranged according to their starting position in the protein sequence, from the N-terminal to the C-terminal.

**Table 5:** IEDB database server-predicted HTL gp120 epitopes.

Length	Peptide	Antigenicity	Allergenicity	Toxicity	Gravy	IC50	IFNepitope
5–19	KLWVTVYYGVPVWKE	−0.2288	No	No	0.147	5.5	Positive
7–21	WVTVYYGVPVWKEAT	0.1562	No	No	0.227	77.1	Positive
16–30	VWKEATTTLFCASDA	−0.0268	No	No	0.267	23.5	Positive
78–92	EDIISLWDQSLKPCV	0.0102	No	No	0.087	9.4	Positive
128–142	NCSFNISTSIRGKVQ	0.6658	No	No	−0.26	10.1	Positive
139–153	GKVQKEYAFFYKLDI	0.7563	No	No	−0.353	21.9	Positive
142–156	QKEYAFFYKLDIIPI	1.3687	No	No	0.147	92.4	Positive
176–190	ACPKVSFEPIPIHYC	1.3979	No	No	0.353	18.4	Positive
202–216	NKTFNGTGPCTNVST	0.2206	No	No	−0.727	11	Positive
228–242	STQLLLNGSLAEEEV	0.2068	No	No	0.067	75.5	Positive
234–248	NGSLAEEEVVIRSVN	0.5764	No	No	−0.087	39.4	Positive
244–258	IRSVNFTDNAKTIIV	1.1445	No	No	0.36	6.9	Positive
261–275	NTSVEINCTRPNNNT	0.9088	No	No	−1.253	36.5	Positive
289–303	FVTIGKIGNMRQAHC	1.1301	No	No	0.14	5.3	Positive
304–318	NISRAKWNNTLKQIA	0.1747	No	No	−0.82	18.1	Positive
383–397	SDTITLPCRIKQIIN	0.3686	No	No	0.107	93	Positive
421–435	ITGLLLTRDGGNSNN	0.5207	No	No	−0.4	7	Positive
450–464	NWRSELYKYKVVKIE	−0.1285	No	No	−0.953	63.9	Positive
459–473	KVVKIEPLGVAPTKA	1.5003	No	No	0.333	17.3	Positive
463–477	IEPLGVAPTKAKRRV	1.4711	No	No	−0.287	21.4	Positive
465–479	PLGVAPTKAKRRVVQ	1.0856	No	No	−0.307	78.9	Positive

The IEDB database server was assessed to anticipate the HTL epitopes. The gp120 epitopes were chosen according to their antigenicity rate, percentage rank, and binding potential to MHC-II HLAs (nM). The SVM approach was used to evaluate each epitope’s potential as an inducer of IFN-γ. For each of the chosen epitopes, the level of conservation was also considered. The potential of the IFN-γ inducer was also compared to other cytokines using a motif and SVM hybrid technique. Based on their varied immunogenic potential, the epitopes were chosen to be a part of the final vaccine formulation. Epitopes in the table are arranged according to their starting position in the protein sequence, from the N-terminal to the C-terminal.

**Table 6:** IEDB database server- predicted HTL Nef epitopes.

Length	Peptide	Antigenicity	Allergenicity	Toxicity	Gravy	IC_50_	IFNepitope
1–15	MGGKWSKSSVIGWPT	0.6597	No	No	−0.327	56	Positive
7–21	KSSVIGWPTVRERMR	1.1439	No	No	−0.753	65	Positive
17–31	RERMRRAEPAADRVG	0.1706	No	No	−1.567	53.6	Positive
28–42	DRVGAASRDLEKHGA	0.4773	No	No	−0.987	82.6	Positive
34–48	SRDLEKHGAITSSNT	0.3181	No	No	−1.08	36.2	Positive
55–69	CAWLEAQEEEEVGFP	0.9747	No	No	−0.467	12.9	Positive
59–73	EAQEEEEVGFPVTPQ	1.0889	No	No	−1.053	65.8	Positive
61–75	QEEEEVGFPVTPQVP	0.8276	No	No	−0.767	57.6	Positive
62–76	EEEEVGFPVTPQVPL	0.7831	No	No	−0.28	13	Positive
63–77	EEEVGFPVTPQVPLR	0.878	No	No	−0.347	8.4	Positive
67–81	GFPVTPQVPLRPMTY	0.7674	No	No	−0.04	81.9	Positive
111–125	DLWIYHTQGYFPDWQ	0.4015	No	No	−0.88	64.5	Positive
118–132	QGYFPDWQNYTPGPG	0.6113	No	No	−1.427	13.2	Positive
177–191	EREVLEWRFDSRLAF	1.0551	No	No	−0.667	74.2	Positive
130–144	GPGVRYPLTFGWCYK	0.9519	No	No	−0.247	25	Positive
142–156	CYKLVPVEPDKIEEA	0.8743	No	No	−0.353	7.9	Positive
185–199	FDSRLAFHHVARELH	0.7962	No	No	−0.36	18.1	Positive
188–202	RLAFHHVARELHPEY	0.9065	No	No	−0.687	9.5	Positive

The IEDB database server was assessed to anticipate the HTL epitopes. Nef epitopes were chosen according to their antigenicity rate, percentage rank, and binding potential to MHC-II HLAs (nM). The SVM approach was used to evaluate each epitope’s potential as an inducer of IFN-γ. For each of the chosen epitopes, the level of conservation was also considered. The potential of the IFN-γ inducer was also compared to other cytokines using a motif and SVM hybrid technique. Based on their varied immunogenic potential, the epitopes were chosen to be a part of the final vaccine formulation. Epitopes in the table are arranged according to their starting position in the protein sequence, from the N-terminal to the C-terminal.

**Table 7:** Various physicochemical characteristics of the vaccine formulation.

Features	Values
Sequence length	757 aa
Molecular weight	83,649.75
Formula	C_3701_H_5680_N_1044_O_1124_S_27_
Antigenicity	0.5819
Theoretical pI	8.87
Total negatively charged residues	74
Total positively charged residues	85
Total number of atoms	11,576
Extinction of coefficients	141,345
Instability index	38.92
AI	59.52
Gravy	−0.646

## Data Availability

Data is contained within the article.
